# Application of atmospheric pressure field desorption for the analysis of anionic surfactants in commercial detergents

**DOI:** 10.1007/s00216-023-04917-y

**Published:** 2023-08-30

**Authors:** Jürgen H. Gross

**Affiliations:** Institute of Organic Chemistry, Heidelberg University, Im Neuenheimer Feld 270, 69120 Heidelberg, Germany

**Keywords:** Atmospheric pressure field desorption (APFD), Field desorption (FD), Atmospheric pressure ionization (API), Activated field emitter, Fourier transform-ion cyclotron resonance (FT-ICR), Accurate mass, Anionic surfactants, Commercial detergents, Negative ions, Ambient desorption/ionization (ADI)

## Abstract

Recent work has shown that field desorption (FD) and field ionization (FI) using activated field emitters may be performed at atmospheric pressure, too. While some limitations apply to atmospheric pressure field desorption (APFD) mass spectrometry (MS), the method can deliver both positive and negative even electron ions of highly polar or ionic compounds. Furthermore, APFD even permits the generation of positive molecular ions of polycyclic aromatic compounds. Here, an application of negative-ion APFD for the analysis of anionic surfactants contained in commercial detergent products for body care, household, and technical uses is presented. The samples include liquid soaps and shower gels, dishwashing liquids, and cooling lubricants. Surfactant solutions in methanol/water or pure methanol at 2–10 µl ml^−1^ were deposited on commercial 13-µm activated tungsten emitters. The emitters were positioned in front of the atmospheric pressure interface of a Fourier transform-ion cyclotron resonance (FT-ICR) mass spectrometer by means of a slightly modified nano-electrospray ionization (nanoESI) source. The entrance electrode of the interface was set to positive high voltage with respect to the emitter at ground potential. Under these conditions, negative-ion desorption was achieved. The surfactant anions, organic sulfates and organic sulfonates, were characterized by accurate mass-based formula assignments, and in part, by tandem mass spectrometry. The negative-ion APFD spectra were compared to results by negative-ion electrospray ionization (ESI) either obtained using the FT-ICR mass spectrometer or by using a trapped ion mobility-quadrupole-time-of-flight (TIMS-Q-TOF) instrument when product ions of low *m/z* needed to be detected in tandem MS.

**Graphical Abstract:**

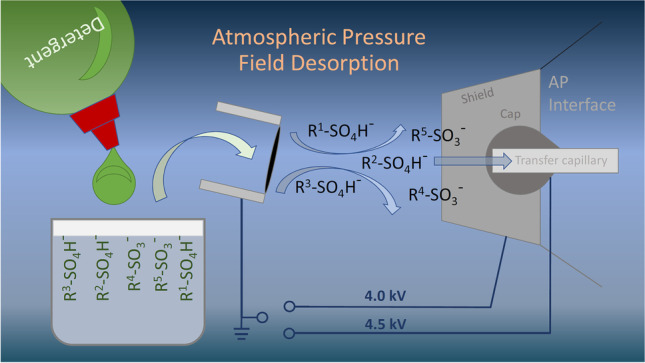

**Supplementary Information:**

The online version contains supplementary material available at 10.1007/s00216-023-04917-y.

## Introduction

The last two decades brought various innovative approaches to ion generation at atmospheric pressure, many of them exploiting the effect of strong electric fields to generate or at least desorb ions from tips, edges, and most importantly sharp pins or needles. Some prominent examples are presented by paper spray ionization [1], tissue paper-assisted spray ionization [2], field-induced wooden tip electrospray ionization [3], the use of a sharp stainless steel needle in front of an API interface [4], and the use of an insulating fiber as sampling probe and ionization substrate [5], particularly carbon fiber ionization [6, 7], and some other techniques [8–13].

Nonetheless, the traditional and longest established soft ionization methods employing strong electric fields in the order of 1–2 V Å^−1^ are field ionization (FI) and field desorption (FD) [14–17]. In contrast to the above techniques, FI and FD are performed in high vacuum. Under these conditions, depending on the polarity of the molecular analytes, FI and FD typically deliver intact positive molecular ions, M^+•^, protonated molecules, [M + H]^+^, and/or alkali ion adducts, [M + alkali]^+^ [14–17]. Ionization via the FI pathway relies on high electric field strengths to effect tunneling of an electron from the neutral analyte molecule towards a positive electrode, i.e., the field emitter [14, 18, 19]. FD of preformed ions on the other side can occur at field strengths being about a hundred times lower [18, 20–24]. The very high electric field strengths required for FI to result in M^+•^ ion formation are achieved by using the so-called activated field emitters [25–27]. Activated tungsten wire emitters provide thousands of field-enhancing microneedles that also serve as a large surface for deposition of the sample. The implementation of FD as liquid-injection field desorption/ionization (LIFDI) [28–33] additionally offers sample application to the emitter under the complete exclusion of moisture and air [30, 31, 34–38].

Especially among those in the community with long experience in FI, FD, and later also LIFDI, it had become manifest that these ionization techniques had to be performed in high vacuum. Nonetheless, the majority of recent mass spectrometers comes with atmospheric pressure interfaces. It therefore deemed advantageous to transfer FD to these platforms. Thus, attempts have been made to permit FD in a non-vacuum environment, preferably at atmospheric pressure. Thus, FD has been performed at superatmospheric pressure (6 bar), thereby allowing for emitter potentials in the order of 10 kV while suppressing electric discharges to the counter electrode [18]. From bare 20-µm tungsten wire emitters set to 9–12 kV relative to a counter electrode at 1.6 or 5.0 mm distance, respectively, positive ions of various ionic and highly polar compounds were generated and FD spectra exhibiting intensive signals were obtained [18].

Activated tungsten field emitters at atmospheric pressure were first tried in a study exploring natural microscale emitters like hairy legs of *Drosophila* flies [39]. There, activated emitters were used as a control experiment, and in fact, [M + H]^+^ ions of hexakis-(fluoroalkoxy)-phosphazenes from a commercial mass calibration mixture [40–42] were detected [39].

Increased reaction rates were observed for three reactions, i.e., a hydrazone formation, a Katritzky reaction, and a Hantzsch synthesis, when standard 13-µm activated tungsten wire emitters were used for FD at the open atmosphere [19]. This phenomenon was attributed to the high electric fields generated by an FD emitter. In this work, the emitters were set to 4–5 kV with respect to the counter electrode at 3–15 mm distance to the orifice of the API interface, typically yielding protonated molecules, [M + H]^+^. Provided high electric field strengths could be applied, and in case of phenylhydrazone, even molecular ions, [C_6_H_8_N_2_]^+•^, were detected [19].

The above studies recently inspired the exploration of atmospheric pressure field desorption (APFD) using standard activated 13-µm tungsten wire emitters [43]. There, the formation of positive and also of negative even electron ions has been reported for ionic or at least highly polar analytes such as ionic liquids, perfluorononanoic acid, polyethylene glycol diacid, and two amino-terminated polypropylene glycols [43]. Negative-ion FD had seldomly been described until then and also had been restricted to high vacuum conditions [36, 44, 45]. Nonetheless, this sort of work already indicated that negative-ion FD was suited to detect anionic surfactants contained in a dishwashing liquid [44].

Subsequently, it was demonstrated that in APFD, even molecular ions, M^+•^, may be formed via field ionization [46]. Thus, molecular ions of various polycyclic aromatic compounds such as benzo[a]pyrene, anthracene, fluoranthene, 1,1,4,4-tetraphenyl-butadiene, and 1-aza-[6]helicene were generated [46].

As, at this stage, the basic characteristics of APFD have been depicted, the present study aims at exploring the capabilities and at identifying potential applications of this new atmospheric pressure variant of FD. This work describes the application of negative-ion APFD for the analysis of anionic surfactants that are common ingredients of various classes of commercial detergents ranging from dishwashing liquids over liquid soaps and shower gels to cooling lubricants.

## Experimental

### Mass spectrometer

All APFD experiments were preformed using a Bruker Apex-Qe Fourier transform-ion cyclotron resonance (FT-ICR) mass spectrometer (Bruker Daltonics, Bremen, Germany) equipped with a 9.4 T superconducting magnet and an ESI-to-MALDI switchable Dual Source. On this instrument, tandem MS can be performed by mass selection of precursor ions in a linear quadrupole (Q) in front of the FT-ICR analyzer. The mass spectrometer was controlled by the Bruker ApexControl software (V 3.0.0) and data analysis was performed using the Bruker DataAnalysis software (V 4.3).

Per transient, ions were collected for 0.5–2.0 s prior to ICR mass analysis in the RF-only accumulation hexapole (h2). Ions were excited and detected using standard settings from previous work [43, 46–48]. External mass calibrations for negative-ion mode were established in ESI mode by either using Agilent Tune Mix (G1969-85000) for the *m/z* 200–1800 range [40–42] or arginine [Arg_n_–H]^−^ cluster ions for the *m/z* 150–1200 range [49–51].

Mass accuracy was generally in the order of 2 ppm. For tandem MS, precursor ions were selected by the quadrupole and activated by collision-induced dissociation (CID) with the argon buffer gas in h2 by applying an offset voltage.

To yield a final FT-ICR mass spectrum, 16 transients were accumulated. When the range *m/z* 200–1800 was selected, a 512 k data points transient resulted in a resolving power of *R* = 72,000 at *m/z* 397, the range *m/z* 150–1200 with an 1 M data points transient delivered *R* = 115,000 at *m/z* 397, respectively. The majority of the APFD and ESI spectra was acquired with the latter settings.

### APFD ion source and operation

The simple APFD setup used and its general operation were described in detail elsewhere [43, 46] and are only briefly summarized here. A custom-built aluminum piece to clamp the FD emitter was mounted onto an *x*,*y*,*z*-adjustable sample stage of the emitter holder of a Bruker nanoESI source. The emitter positioning process could be observed using the built-in CCD camera and a monitor. The experiments described here used what was described as C2 in [43], i.e., the conventional ESI interface with the so-called spray shield and a rounded metal cap (orifice 0.90 mm in diameter) mounted on the glass transfer capillary (orifice 0.50 mm in diameter) underneath (Supplementary Material Figs. S1 and S2).

High voltage was exclusively applied to the counter electrode provided by the API interface while the emitter holder inside the nanoESI source stayed at ground potential. The high voltage was set using the API source controls. At the typical distance of the emitter to the counter electrode, in this setup represented by the spray shield and capillary cap, voltages close to the upper technical limit of the source (+ 6 kV) normally caused spark discharges. Thus, the voltages in the range of + 3.0 to + 4.8 kV at the spray shield and + 3.5 to + 5.5 kV at the cap underneath the spray shield were employed.

As the ApexControl software (V 3.0.0) did not permit to alter the high voltage during an acquisition, it was mandatory to first increase the voltages until desorption was observed in the so-called tune mode of the ApexQe instrument, before switching to acquisition mode. During this switching period taking 15–20 s ion desorption inevitably continued without contributing to the spectrum, i.e., a notable portion of the sample was wasted, thereby leading to a decrease in overall sensitivity of the method.

The nebulizer gas for ESI was switched off at all times, and the drying gas was either off or set to 1.2–2.0 l min^−1^ at 100–140 °C. Other instrument settings were the same as in ESI operation.

The samples were manually delivered to the emitter as solutions in methanol: water = 9:1 or pure methanol at concentrations of 2–10 µl ml^−1^ by using a 10-µl syringe while the emitter was clamped into the emitter holder. The solvents were allowed to evaporate and the emitter was then mounted to the nanoESI source. After the runs, the emitter was rinsed with solvent to remove excessive analyte. Emitters could be used for tens of acquisitions.

The activated field emitters were of the standard type commercially available for the JEOL AccuTOF series of instruments [44, 52] and were based on 13-µm tungsten wires (Linden CMS, Weyhe, Germany).

Control experiments were performed by negative-ion electrospray ionization (ESI) using the same FT-ICR mass spectrometer and operated as previously described [48, 53]. For ESI, the solution for APFD was diluted in methanol by a factor of 20–50. In addition, a Bruker timsTOFflex, i.e., a trapped ion mobility-quadrupole-time-of-flight (TIMS-Q-TOF) instrument (Bruker Daltonics, Bremen, Germany), was used when product ions of low *m/z* needed to be detected in tandem mass spectra to control the structural assignment of the analytes.

### Detergent samples

The detergent samples of various types are compiled in Table 1. Dishwashing liquids and body care products were collected from bottles at the author’s affiliation and home, respectively. All products were obtained from German stores. The cooling lubricants were collected from the institute’s mechanics workshop.

**Table 1:** Compilation of the detergent samples analyzed

Product category	Product type	Product name
Household	Dishwashing liquid	Pril Kraftgel
Household	Dishwashing liquid	Fairy Ultra
Household	Dishwashing liquid	Fairy Ultra Plus
Household	Windscreen anti-frost liquid	Robbyrob Scheibenfrostschutz
Body care	Liquid soap	Today Seife Aqua
Body care	Liquid soap	Today Seife Sensitive
Body care	Liquid soap	Balea Milde Seife
Body care	Shower gel	Ombia Duschgel
Body care	Shower gel	Dusy Women Duschgel
Technical	Cooling lubricant	BlasoCut
Technical	Cooling lubricant	AluFluid

In addition, the ionic liquid (IL) trihexyl(tetradecyl)phosphonium tris(pentafluoroethyl)-trifluorophosphate) (Merck KGaA, Darmstadt, Germany) delivering a very intensive anion signal at *m/z* 444.9456 (calc. for [C_6_F_18_P]^−^) was used to test APFD operation and general FT-ICR instrument settings in MS and tandem MS mode before analyzing the detergents (Fig. S3) [43].

## Results and discussion

### General characteristics of surfactant spectra

**Fig. 1:**
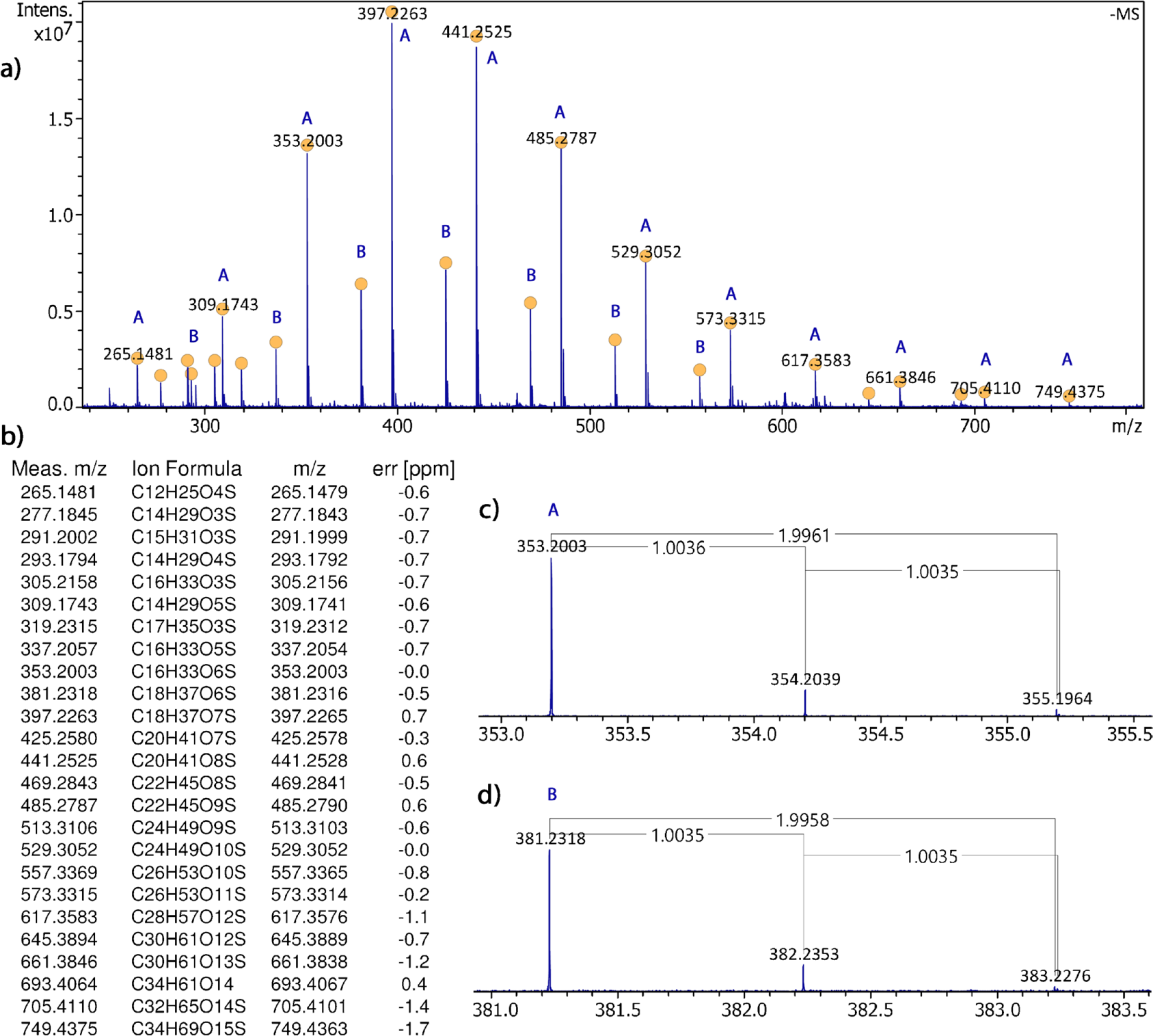
In **a**, the negative-ion APFD spectrum of Pril Kraftgel shows two major series of signals marked with A and B that are corresponding to homologous ions. **b** The formulas assigned by accurate mass are supplied in the list along with the calculated *m/z* values and relative errors. Yellow dots at the peak tops mark peaks with formula assignments in the list below the spectral plot. The expanded views of the signals **c** at *m/z* 353.2003 and **d** *m/z* 381.2318 reveal the presence of sulfur in members of both ion series due to the doublet peaks caused by the respective ^13^C_2_ and ^34^S isotopic ions

As mentioned before, a study of negative-ion FD already indicated that this technique was suited to detect anionic surfactants contained in a dishwashing liquid [44]. In addition, during a recent exploration of APFD, it appeared that negative-ion APFD should also be suited for that type of analytes [43]. As the dishwashing liquid Pril Kraftgel was the only detergent tested in negative-ion FD so far [44], it was deemed reasonable to begin in APFD with same. The negative-ion APFD spectrum of Pril Kraftgel exhibits two major series of signals (Fig. 1). Series A starts at nominal *m/z* 265 and leads up to *m/z* 749 in steps of 44 u while series B starts at nominal *m/z* 293 and leads to *m/z* 693, also in increments of 44 u. Upon zooming in at the isotopic patterns, all of these signals reveal the presence of one sulfur atom at the M + 2 peaks due to separation of the ^13^C_2_ ions (Δ(*m/z*)_calc_ = 2 × 1.0033) and the ^34^S ions (Δ(*m/z*)_calc_ = 1.9958). Combined with accurate mass data, the formulas of the corresponding ions can be assigned. The first ones of series A are [C_12_H_25_O_4_S]^−^ (calc. 265.1479), [C_14_H_29_O_5_S]^−^ (calc. 309.1741), [C_16_H_33_O_6_S]^−^ (calc. 353.2003), and [C_18_H_37_O_7_S]^−^ (calc. 397.2265), the latter being the most abundant ion. For series B, one obtains [C_14_H_29_O_4_S]^−^ (calc. 293.1792), [C_16_H_33_O_5_S]^−^ (calc. 337.2054), [C_18_H_37_O_6_S]^−^ (calc. 381.2316), and [C_20_H_41_O_7_S]^−^ (calc. 425.2578), also with the latter being the most abundant one (Fig. 1). Either series obviously starts with a saturated aliphatic sulfate, indicating fatty acid alcohol sulfate structures. The difference to the next higher member of each ion series corresponds to C_2_H_4_O units, and thus, points towards a polyethyleneglycol unit being part of the structure of these ions. Comparing the negative-ion APFD spectrum to a negative-ion ESI spectrum as the reference, shows that both are very close in appearance, i.e., they exhibit peaks due to the same major ion series, and moreover, are very similar in their intensity distribution. A pair of a different APFD spectrum and an ESI spectrum is provided in Figs. S4 and S5. This comparison (and further examples later in this paper) demonstrates that negative-ion APFD of the dishwashing liquid yields the same ion series, intensity distribution, and corresponding ionic formulas. In other words, the ESI spectra serve as a validation of APFD data, and vice versa, APFD essentially delivers information equivalent to the well-proven ESI technique.

### Tandem MS for structure elucidation

When APFD tandem mass spectra were acquired using the ApexQe FT-ICR instrument, upon CID, a notable decrease in intensity of the precursor ion peak did not go along with the appearance of fragment ion signals within the accessible *m/z* range. This behavior was experienced with several precursor ions and has been exemplified in case of a precursor ion at *m/z* 397.2 (Fig. S6). When the precursor ion *m/z* 397.2 was selected at a collision offset voltage of just 1 V or 10 V, i.e., as long as fragmentation did not play a role, the intensity of this signal remained in the order of 1 × 10^7^ counts. Increasing the collision offset voltage to 20 V caused a drop to 6.0 × 10^5^ counts. Even though the precursor ion peak essentially disappeared, no fragment ion peaks were found within the accessible *m/z* range. This was interpreted in terms of the formation of fragment ions below *m/z* 170, which is the low-mass cut-off of this particular instrument.

In addition, like classical FD spectra, APFD spectra are only acquired during a short period of time, normally 10–60 s, while continuous sample supply from a syringe pump can deliver almost constant ion current as long as required to collect a larger set of tandem mass spectra. As the same ions were observed in negative-ion APFD and ESI, the ESI mode was finally chosen for tandem MS.

**Fig. 2:**
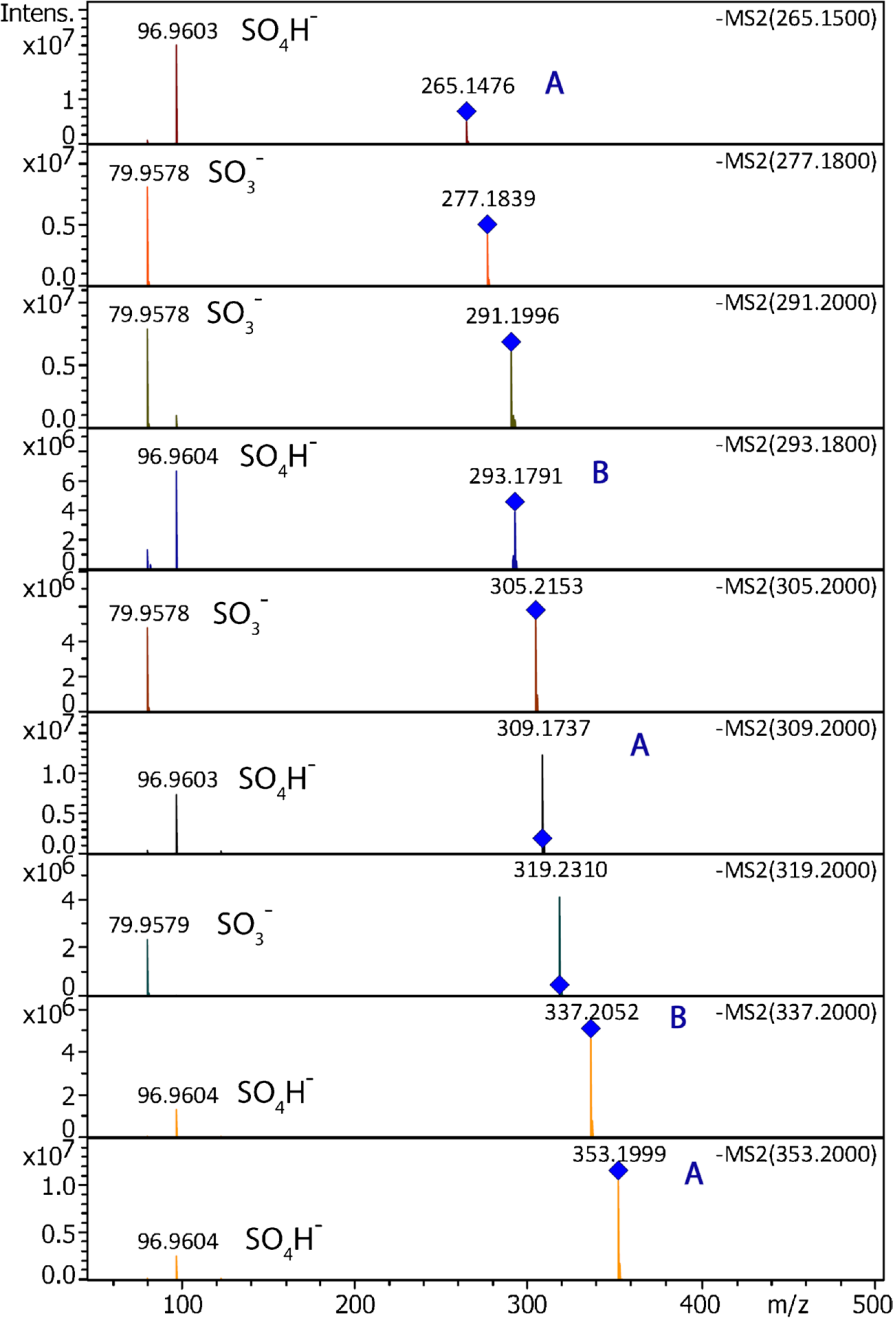
Negative-ion ESI tandem mass spectra of nine precursor ions selected from Pril Kraftgel as obtained using the trapped ion mobility-quadrupole-time-of-flight (TIMS-Q-TOF) instrument in order to cover product ions of low *m/z*. The precursor ion peaks are marked by a blue diamond and the *m/z* values are also denoted at the top right of each spectrum. The collision offset was 30 V for all precursor ions, some of which fragment to yield [SO_3_]^−^ (calc. 79.9574 u) as the only product ion, others to form [SO_4_H]^−^ (calc. 96.9601 u). Members of series A and B are marked

Furthermore, because the fragment ions were expected below *m/z* 170, the analyses were repeated on a timsTOFflex instrument, that without using the ion mobility separation served as a Q-TOF having tandem MS characteristics basically equal to the ApexQe FT-ICR mass spectrometer. A set of precursor ions covering the smaller members of series A and B plus some other ions located between. Under these conditions (collision offset was 30 V for all precursor ions), fragment ions at nominal *m/z* 80 and *m/z* 97 appeared as the only fragment ion species formed of any of these ions (Fig. 2). Based on their accurate mass, these fragment ions were assigned to [SO_3_]^−^ (calc. 79.9574 u) and to [SO_4_H]^−^ (calc. 96.9601 u), respectively. Thus, the tandem mass spectra confirmed the assumption that the surfactant anions either belong to organic sulfates (*m/z* 265, 293, 309, 337, 353) or sulfonates (*m/*z 277, 291, 305, 319), respectively. More detailed information on the position of poly-ethyleneglycol moieties was not available as there were no fragment ions related to this part of the molecules.

### Other detergents

**Fig. 3:**
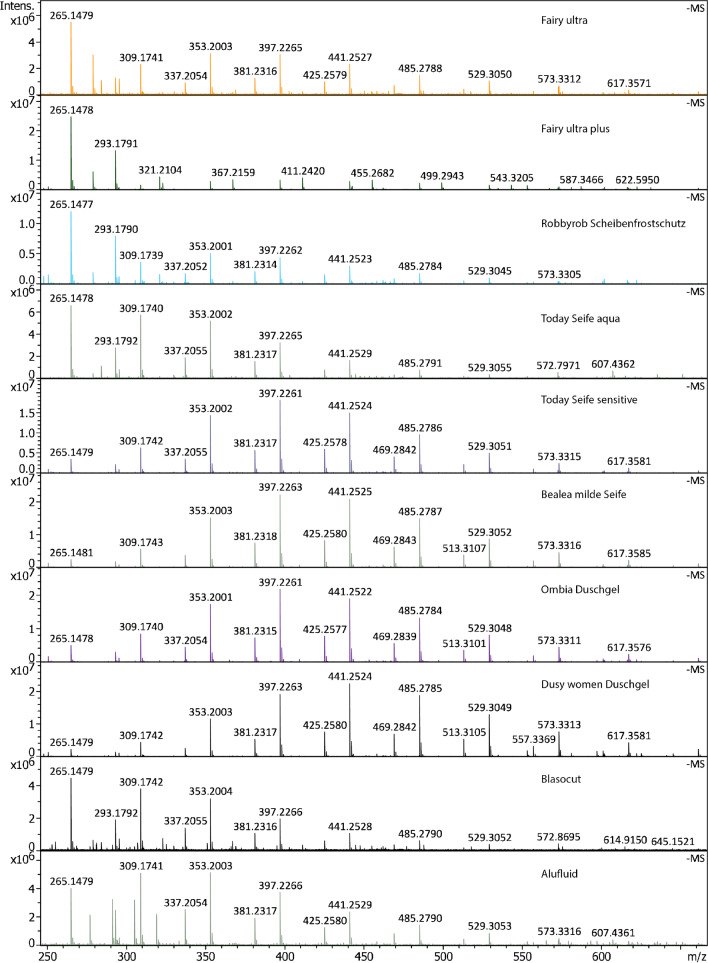
Compilation of negative-ion APFD spectra revealing anionic surfactants as contained in a selection of ten commercial detergents. While the *m/z* 150–1200 range was acquired, the section shown here covers the relevant range

To substantiate the finding that negative-ion APFD can be used for the analysis of anionic surfactants in commercial detergents, the method was next applied to ten additional samples, some other dishwashing liquids, an anti-frost windscreen washing liquid, body care products like liquid soaps and shower gels, and even to cooling lubricants used for machining of metals (Table 1). The negative-ion APFD spectra revealing the anionic surfactants as contained in these ten commercial detergents are summarized in Fig. 3. In addition, each of these APFD spectra along with formula lists and a comparison to a negative-ion ESI spectrum are collected in the Supplementary Material as Figs. S7 to S26. As can be seen, all of these commercial products contain quite similar amounts of anionic surfactants that mostly belong to the same classes as described above. While there are some variations in molecular weight distribution, the majority of the compounds detected by APFD is equal across all kinds of products. A closer look indicates two different formulations, maybe due to different suppliers or due to intentional selection for the specific purpose, one leaning to the low-mass side with *m/z* 265 or *m/z* 309 as the base peak, the other being centered around *m/z* 397 or *m/z* 441 as the base peak. Among these, Today Seife aqua has been taken as another candidate for tandem MS of a series of precursor ions (*m/z* 265, 293, 309, 337, 353, 381, 397, 425, 441) using the timsTOFflex instrument. Unsurprisingly, the resulting spectra all corresponded to organic sulfates (Fig. S14).

In the present context, the results shall be taken as a proof of the ability of negative-ion APFD to allow for the detection of anionic surfactants in a large variety of commercial detergent products that contain additional compound classes in order to achieve the overall properties required for the respective application. Cooling lubricants, for example, contain mineral oil and/or vegetable oil, the latter being also of interest in body care products.

### Sample consumption

Comparing APFD and ESI spectra provided in the Supplementary Material reveals that not only the ion series but also the absolute intensities of the signals are comparable, e.g., the base peak of the APFD spectrum of Pril Kraftgel appears at around 2.5 × 10^7^ counts while that of the ESI spectrum appears at 3.2 × 10^7^ counts (Fig. 1 and Figs. S4, S5). The APFD spectrum was acquired for 16 × 1.5 s while the ESI spectrum was obtained from 16 × 0.5 s using a 25-fold diluted solution. ESI thus turns out to be about 80 times more sensitive than APFD. While this may appear discouraging at first sight, one has to take into account that vacuum FD generally delivers very low ion currents, and therefore, this difference may be regarded as a promising level for APFD to start with.

The minimum sample consumption for an APFD spectrum was determined by stepwise dilution of the detergent solution, in this case using dusy women shower gel (Fig. S27). Spectra acquired using 1 µl of solution at 10 µl ml^−1^ typically resulted in maximum intensities of about 2 × 10^7^ counts (Figs. S6 and S22), spectra acquired at 1.0 µl ml^−1^ were found to deliver around 4 × 10^6^ counts, and those at 0.1 µl ml^−1^ still yielded similar results. At 0.01 µl ml^−1^, the spectra essentially vanished. Such threshold behavior was also observed on other occasions with the ApexQe instrument. Thus, the minimum sample amount to reliably deliver a useful APFD spectrum was in the order of 0.1 µg of detergent. Assuming the anionic surfactants to contribute about 20% of the detergent, this corresponds to 0.02 µg of anionic surfactants required for an APFD spectrum.

## Conclusion

Negative-ion APFD has been applied to the analysis of anionic surfactants contained in a number of commercial detergents ranging from body care products like liquid soaps and shower gels over dishwashing liquids to cooling lubricants. The spectra were generally in line with those obtained by negative-ion ESI performed as a control experiment. In APFD, the anionic surfactants, essentially organic sulfonates and sulfates, contained as major active ingredients were found to deliver anions that were typically detected across the *m/z* 250–750 range. Surprisingly, across all of these different products, two major series of anion signals appeared. The findings also reveal two molecular weight distributions to be used, independent of whether the product aimed at body care, household, or technical use. Nonetheless, the data presented here is not meant to deliver a complete analysis of the various commercial detergents or to evaluate their quality for the customer but solely to explore the capabilities and potential applications of APFD-MS.

Admittedly, in its present state, the new approach of APFD does not offer an advantage over the long-established and highly optimized ESI technique for this sort of application. Nevertheless, the potential of any new method needs to be explored. This is an important and inevitable step in the development of any ionization technique. An easier to use and more robust device for better reproducibility of emitter positioning may be expected to contribute to a more effective consumption of sample, and eventually, to result in a more competitive technique.

### Supplementary Information

Below is the link to the electronic supplementary material.

Supplementary file1 (PDF 10396 KB)

## References

[1] Liu J, Wang H, Manicke NE, Lin J-M, Cooks RG, Ouyang Z (2010). Development, characterization, and application of paper spray ionization. Anal Chem.

[2] Meher AK, Chen Y-C (2015). Tissue paper assisted spray ionization mass spectrometry. RSC Adv.

[3] Yang Y, Deng J, Yao Z-P (2015). Field-induced wooden-tip electrospray ionization mass spectrometry for high-throughput analysis of herbal medicines. Anal Chim Acta.

[4] Hiraoka K, Nishidate K, Mori K, Asakawa D, Suzuki S (2007). Development of probe electrospray using a solid needle. Rapid Commun Mass Spectrom.

[5] Selvaprakash K, Chen Y-C (2022). Using an insulating fiber as the sampling probe and ionization substrate for ambient ionization–mass spectrometric analysis of volatile, semi-volatile, and polar analytes. Anal Bioanal Chem.

[6] Wu M-L, Chen T-Y, Chen Y-C, Chen Y-C (2017). Carbon fiber ionization mass spectrometry for the analysis of analytes in vapor, liquid, and solid phases. Anal Chem.

[7] Wu M-L, Wu Y-C, Chen Y-C (2019). Detection of pesticide residues on intact tomatoes by carbon fiber ionization mass spectrometry. Anal Bioanal Chem.

[8] Kuo C-P, Shiea J (1999). Application of direct electrospray probe to analyze biological compounds and to couple to solid-phase microextraction to detect trace surfactants in aqueous solution. Anal Chem.

[9] Hong C-M, Lee C-T, Lee Y-M, Kuo C-P, Yuan C-H, Shiea J (1999). Generating electrospray from solutions predeposited on a copper wire. Rapid Commun Mass Spectrom.

[10] Kuo C-P, Yuan C-H, Shiea J (2000). Generation of electrospray from a solution predeposited on optical fibers coiled with a platinum wire. J Am Soc Mass Spectrom.

[11] Jeng J, Shiea J (2003). Electrospray ionization from a droplet deposited on a surface-modified glass rod. Rapid Commun Mass Spectrom.

[12] Jeng J, Lin C-H, Shiea J (2005). Electrospray from nanostructured tungsten oxide surfaces with ultralow sample volume. Anal Chem.

[13] Feider CL, Krieger A, DeHoog RJ, Eberlin LS (2019). Ambient ionization mass spectrometry: recent developments and applications. Anal Chem.

[14] Beckey HD (1971). Field-ionization mass spectrometry.

[15] Beckey HD (1977). Principles of field desorption and field ionization mass spectrometry.

[16] Prókai L (1990). Field desorption mass spectrometry.

[17] Gross JH (2020). From the discovery of field ionization to field desorption and liquid injection field desorption/ionization-mass spectrometry—a journey from principles and applications to a glimpse into the future. Eur J Mass Spectrom.

[18] Chen LC, Rahman MM, Hiraoka K (2012). Non-vacuum field desorption ion source implemented under super-atmospheric pressure. J Mass Spectrom.

[19] Chen X, Cooks RG (2018). Accelerated reactions in field desorption mass spectrometry. J Mass Spectrom.

[20] Heinen HJ, Giessmann U, Röllgen FW (1977). Field desorption of electrolytic solutions using untreated wire emitters. Org Mass Spectrom.

[21] Veith HJ (1977). Alkali ion addition in FD mass spectrometry Cationization and protonation-ionization methods in the application of nonactivated emitters. Tetrahedron.

[22] Röllgen FW, Giessmann U, Borchers F, Levsen K (1978). Collisional activation spectra of [M+Li]^+^, [M+Na]^+^ and [M+K]^+^ ions formed by field desorption of some monosaccharides. Org Mass Spectrom.

[23] Keough T, DeStefano AJ (1981). Acid-enhanced field desorption mass spectrometry of zwitterions. Anal Chem.

[24] Davis SC, Neumann GM, Derrick PJ (1987). Field desorption mass spectrometry with suppression of the high field. Anal Chem.

[25] Beckey HD, Hilt E, Schulten H-R (1973). High temperature activation of emitters for field ionization and field desorption spectrometry. J Phys E: Sci Instrum.

[26] Linden HB, Hilt E, Beckey HD (1978). High-rate growth of dendrites on thin wire anodes for field desorption mass spectrometry. J Phys E: Sci Instrum.

[27] Rabrenovic M, Ast T, Kramer V (1981). Alternative organic substances for generation of carbon emitters for field desorption mass spectrometry. Int J Mass Spectrom Ion Phys.

[28] Linden HB. Quick soft analysis of sensitive samples under inert conditions by in-source liquid injection FD In ASMS. Orlando: poster MPL. 2002;373.

[29] Schaub TM, Hendrickson CL, Qian K, Quinn JP, Marshall AG (2003). High-resolution field desorption/ionization Fourier transform ion cyclotron resonance mass analysis of nonpolar molecules. Anal Chem.

[30] Linden HB (2004). Liquid injection field desorption ionization: a new tool for soft ionization of samples including air-sensitive catalysts and non-polar hydrocarbons. Eur J Mass Spectrom.

[31] Gross JH, Nieth N, Linden HB, Blumbach U, Richter FJ, Tauchert ME, Tompers R, Hofmann P (2006). Liquid injection field desorption/ionization of reactive transition metal complexes. Anal Bioanal Chem.

[32] Linden HB, Gross JH (2011). A liquid injection field desorption/ionization-electrospray ionization combination source for a Fourier transform ion cyclotron resonance mass spectrometer. J Am Soc Mass Spectrom.

[33] Linden HB, Gross JH (2012). Reduced fragmentation in liquid injection field desorption/ionization-Fourier transform ion cyclotron resonance mass spectrometry by use of helium for the thermalization of molecular ions. Rapid Commun Mass Spectrom.

[34] Langlotz BK, Fillol JL, Gross JH, Wadepohl H, Gade LH (2008). Living radical polymerization of acrylates mediated by 1,3-bis(2-pyridylimino)isoindolatocobalt(II) complexes: monitoring the chain growth at the metal. Chem Eur J.

[35] Muhr M, Heiß P, Schütz M, Bühler R, Gemel C, Linden MH, Linden HB, Fischer RA (2021). Enabling LIFDI-MS measurements of highly air sensitive organometallic compounds: a combined MS/glovebox technique. Dalton Trans.

[36] Taubert J, Vogt M, Langer R (2023). Mass spectrometric detection of ion pairs containing rigid copper clusters and weakly coordinating counter ions using liquid injection field desorption/ionisation. Eur J Mass Spectrom.

[37] Lainer T, Fischer RC, Haas M (2023). Identification and characterization of selected silyl substituted silyl anions by liquid injection field desorption ionization mass spectrometry. Eur J Mass Spectrom.

[38] Linden MH, Linden HB (2023). Unprecedented intact radical anions, closed shell anions, cluster ions, and traditional cations and radical cations by LIFDI–MS. Eur J Mass Spectrom.

[39] Pirkl A, Dreisewerd K, Yew JY, König S (2010). Field-based ion generation from microscale emitters on natural and artificial objects for atmospheric pressure mass spectrometry. Anal Bioanal Chem.

[40] Moini M (1994). Ultramark 1621 as a calibration/reference compound for mass spectrometry II Positive- and negative-ion electrospray ionization. Rapid Commun Mass Spectrom.

[41] Flanagan JM. Mass spectrometry calibration using homogeneously substituted fluorinated triazatriphosphorines. US Patent. 1997, pat. No. 5872357.

[42] Wu J, McAllister H (2003). Exact mass measurement on an electrospray ionization time-of-flight mass spectrometer: error distribution and selective averaging. J Mass Spectrom.

[43] Gross JH (2023). Desorption of positive and negative ions from activated field emitters at atmospheric pressure. Eur J Mass Spectrom.

[44] Linden MH, Linden HB, Gross JH (2021). Negative-ion field desorption revitalized by using liquid injection field desorption/ionization-mass spectrometry on recent instrumentation. Anal Bioanal Chem.

[45] Drusgala M, Linden MH, Knoechl A, Torvisco A, Fischer RC, Bernhard Linden H (2021). Haas M (2021) Synthesis, LIFDI mass spectrometry and reactivity of triacyl-germenolates. Eur J Inorg Chem.

[46] Hoyer M, Gross JH (2023). Molecular ion formation on activated field emitters in atmospheric pressure field desorption mass spectrometry. Anal Bioanal Chem.

[47] Gross JH (2013). Polydimethylsiloxane-based wide range mass calibration for direct analysis in real time mass spectrometry. Anal Bioanal Chem.

[48] Gross JH (2015). Analysis of silicones released from household items and baby articles by direct analysis in real time-mass spectrometry. J Am Soc Mass Spectrom.

[49] Zhang D, Wu L, Koch KJ, Cooks RG (1999). Arginine clusters generated by electrospray ionization and identified by tandem mass spectrometry. Eur Mass Spectrom.

[50] Soga T, Kakazu Y, Robert M, Tomita M, Nishioka T (2004). Qualitative and quantitative analysis of amino acids by capillary electrophoresis-electrospray ionization-tandem mass spectrometry. Electrophor.

[51] Schug K, Lindner W (2004). Development of a screening technique for noncovalent complex formation between guanidinium- and phosphonate-functionalized amino acids by electrospray ionization ion trap mass spectrometry: assessing ionization and functional group interaction. Int J Mass Spectrom.

[52] Linden MH, Linden HB, Nieth N, Gross JH (2019). Self-supplied liquid injection field desorption/ionization ion source for an orthogonal time-of-flight instrument. J Am Soc Mass Spectrom.

[53] Gross JH (2014). High-mass cluster ions of ionic liquids in positive-ion and negative-ion DART-MS and their application for wide range mass calibrations. Anal Bioanal Chem.

