# Supplementary material for: Application of atmospheric pressure field desorption for the analysis of anionic surfactants in commercial detergents
Source: Anal Bioanal Chem. 2023 Aug 30;415(26):6421–30. doi: 10.1007/s00216-023-04917-y (PMC10567867; doi:10.1007/s00216-023-04917-y)

Application of atmospheric pressure field desorption for the analysis of anionic surfactants in commercial detergents

Supplementary Data

Jürgen H. Gross (ORCID 0000-0003-0748-2535)

Address

Institute of Organic Chemistry

Heidelberg University

Im Neuenheimer Feld 270

69120 Heidelberg

Germany

Correspondence to

email: juergen.gross@oci.uni-heidelberg.de

phone: +49/6221/54-8409

Fig. S1. a) Instrumental set-up when using the Bruker nanoESI source for atmospheric pressure field desorption (APFD). **b)** Custom-built emitter holder. **c)** By opening the small clip on the right side, the dark part of the source can swing open around a hinge. **d)** Access to the emitter holder between the CCD cameras.

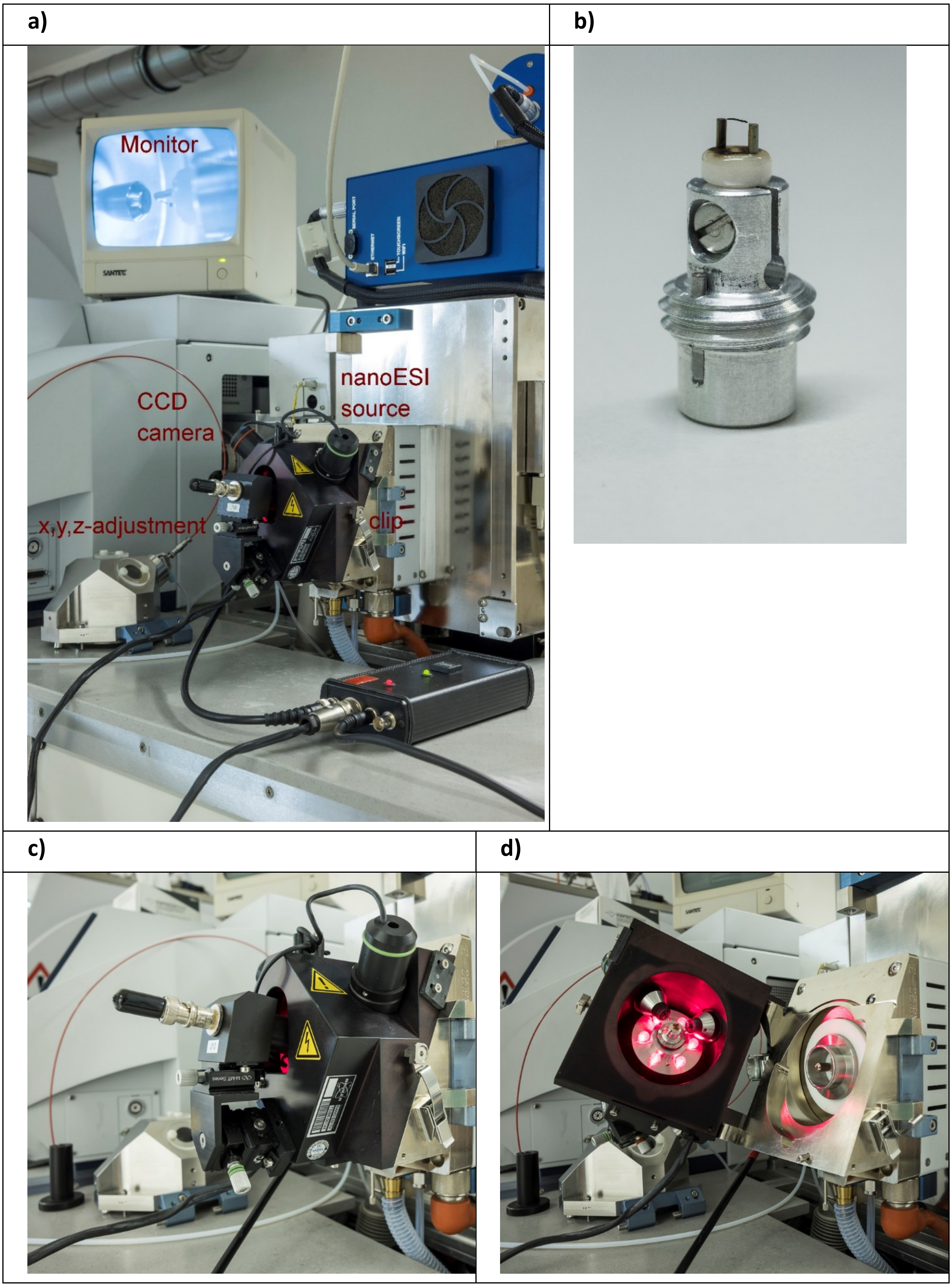

Also cf. Suppl. Gross J. H. *Eur J Mass Spectrom* 2023; 29: 21-32. DOI: 10.1177/14690667221133388

Fig. S2. Photographs of (*top*) the monitor display delivered by the built-in CCD camera of the nanoESI source and (*bottom*) the actual set-up as seen via the right camera port of the nanoESI source. The conventional API interface part with complete spray shield and metal cap on the transfer capillary underneath it remains in the same configuration as used for ESI.

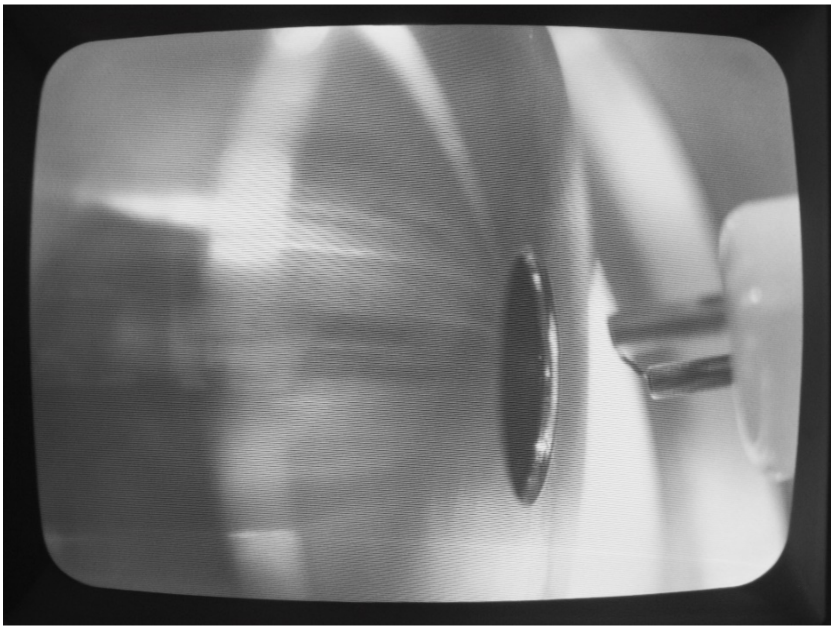

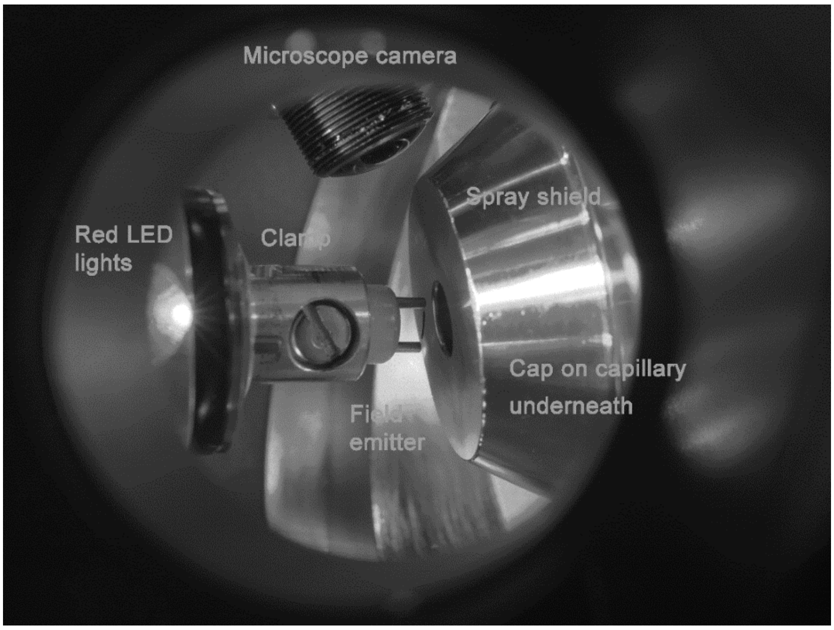

Also cf. Hoyer M and Gross J. H. *Anal Bioanal Chem* 2023; 415: 2307-2315. DOI: 10.1007/s00216-023-04652-4 and Supplementary Material and Gross J. H. *Eur J Mass Spectrom* 2023; 29: 21-32. DOI: 10.1177/14690667221133388 and Supplementary Material.

Fig. S3. Negative-ion APFD spectra of ionic liquid (IL) trihexyl(tetradecyl)phosphonium tris(pentafluoroethyl)trifluorophosphate) as obtained when the IL was used for testing of the instrument prior to analysis of the detergents. (*Top*) The IL anion, $[C_6F_{18}P]^-$, *m/z* 444.9452 (calc. 444.9456) was normally observed at very high intensity, here 2.6×10^7 counts with spray shield +4.0 kV and cap +4.5 kV, desolvation gas flow of 1.2 l min^{-1} at 140 °C. (*Bottom*) The IL anion was also used to check the tandem MS settings. The tandem mass spectrum of the anion at a collision offset of 25 V showed a fragment ion at *m/z* 344.9519 that could be assigned to the formula $[C_4F_{14}P]^-$ (calc. 344.9520) by loss of C_2F_4. The blue diamond marks the precursor ion.

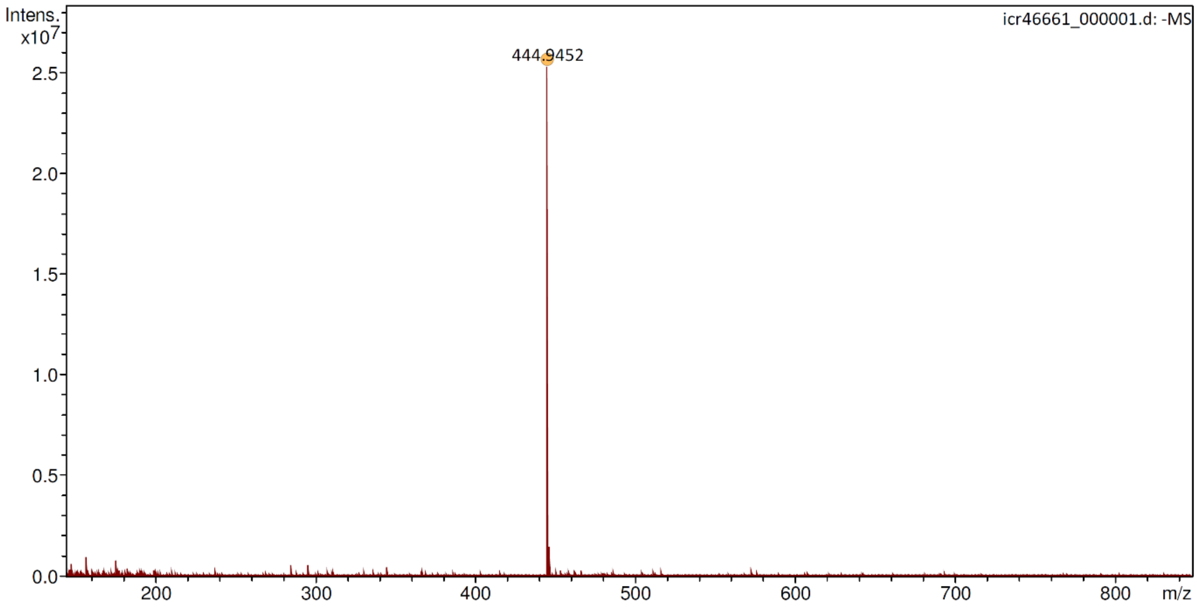

Meas. m/z	Ion Formula	m/z	err [ppm]	mSigma	rdb	e⁻ Conf
444.9452	C6F18P	444.9456	0.9	3.2	-1.5	even

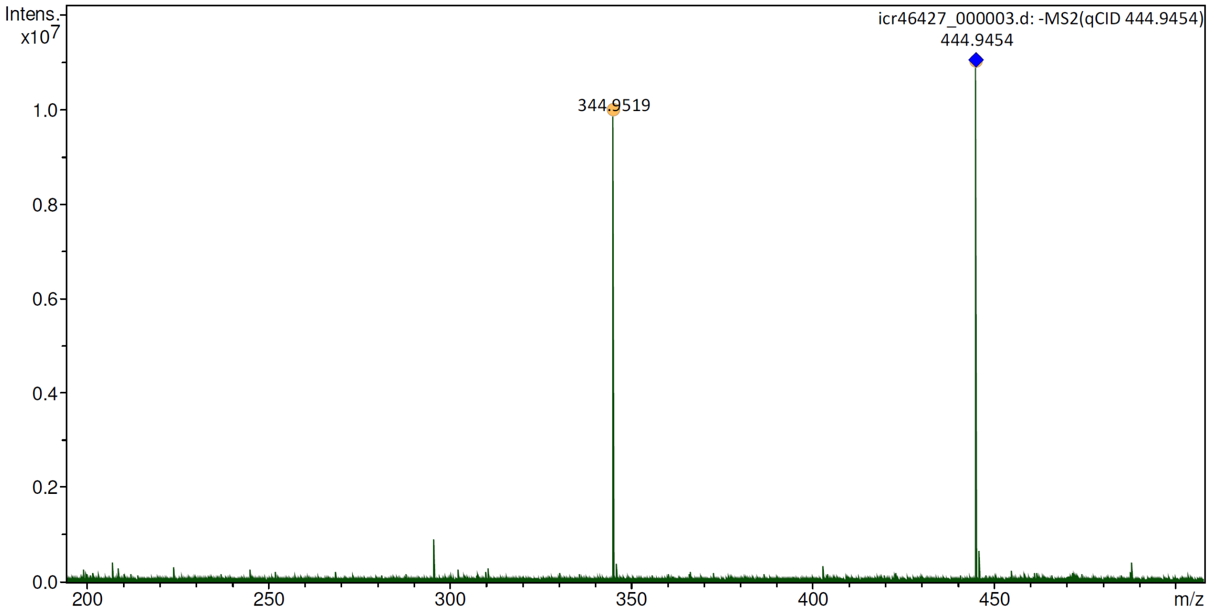

Meas. m/z	Ion Formula	m/z	err [ppm]	mSigma	rdb	e⁻ Conf
344.9519	C4F14P	344.9520	0.2	2.8	-1.5	even
444.9454	C6F18P	444.9456	0.4	1.9	-1.5	even

Fig. S4. Negative-ion APFD spectrum of Pril Kraftgel dishwashing liquid. APFD conditions: 1–2 µl of a solution at 3 µl ml^{-1} in methanol : water = 9 : 1, acquisition 16 × 1.5 s, desolvation gas 1.2 l min^{-1} at 140 °C, shield 3.5 kV, cap 4.0 kV. The formulas assigned by accurate mass are supplied in the list along with the calculated *m/z* values and relative error. Yellow dots at the peak tops mark peaks with formula assignments in the list below the spectral plot.
The expanded views of the signals at *m/z* 353.2000 and *m/z* 381.2314 reveal the presence of sulfur in members of both ion series due to the doublet peaks caused by the respective $^{13}C_2$ and ^{34}S isotopic ions.

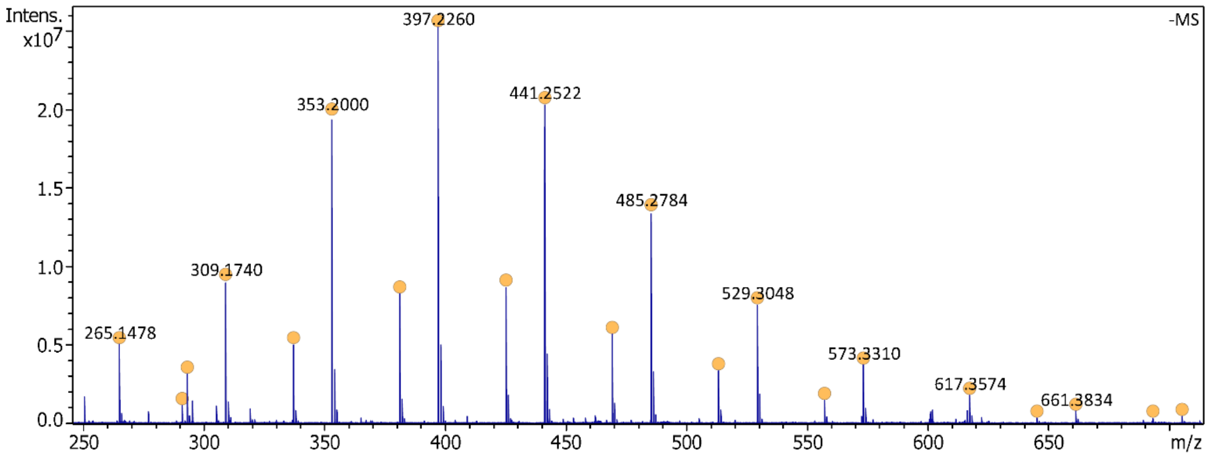

Meas. m/z	Ion Formula	m/z	err [ppm]
265.1478	C12H25O4S	265.1479	0.3
291.1999	C15H31O3S	291.1999	0.2
293.1791	C14H29O4S	293.1792	0.2
309.1740	C14H29O5S	309.1741	0.4
337.2053	C16H33O5S	337.2054	0.3
353.2000	C16H33O6S	353.2003	0.9
381.2314	C18H37O6S	381.2316	0.5
397.2260	C18H37O7S	397.2265	1.3
425.2576	C20H41O7S	425.2578	0.5
441.2522	C20H41O8S	441.2528	1.3
469.2838	C22H45O8S	469.2841	0.6
485.2784	C22H45O9S	485.2790	1.2
513.3100	C24H49O9S	513.3103	0.5
529.3048	C24H49O10S	529.3052	0.8
557.3362	C26H53O10S	557.3365	0.6
573.3310	C26H53O11S	573.3314	0.7
617.3574	C28H57O12S	617.3576	0.4
645.3883	C30H61O12S	645.3889	0.9
661.3834	C30H61O13S	661.3838	0.7
693.3380	C29H57O16S	693.3373	-1.0
705.4099	C32H65O14S	705.4101	0.2

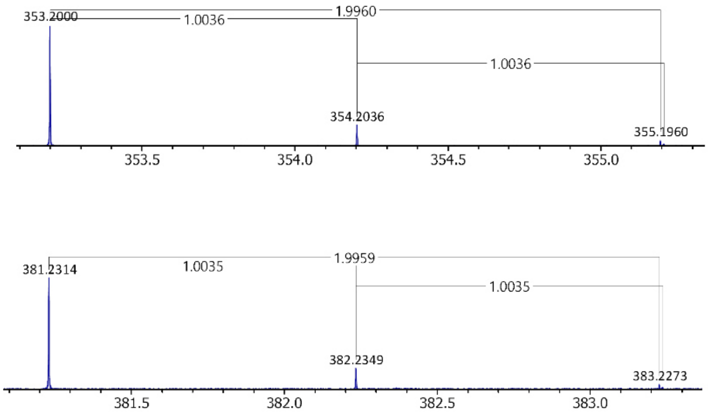

Fig. S5. Negative-ion ESI spectrum of Pril Kraftgel dishwashing liquid. ESI conditions: 6 µl min^{-1} of a solution at 0.1 µl ml^{-1} in methanol : water = 9 : 1. Yellow dots at the peak tops mark peaks with formula assignments in the list below the spectral plot.

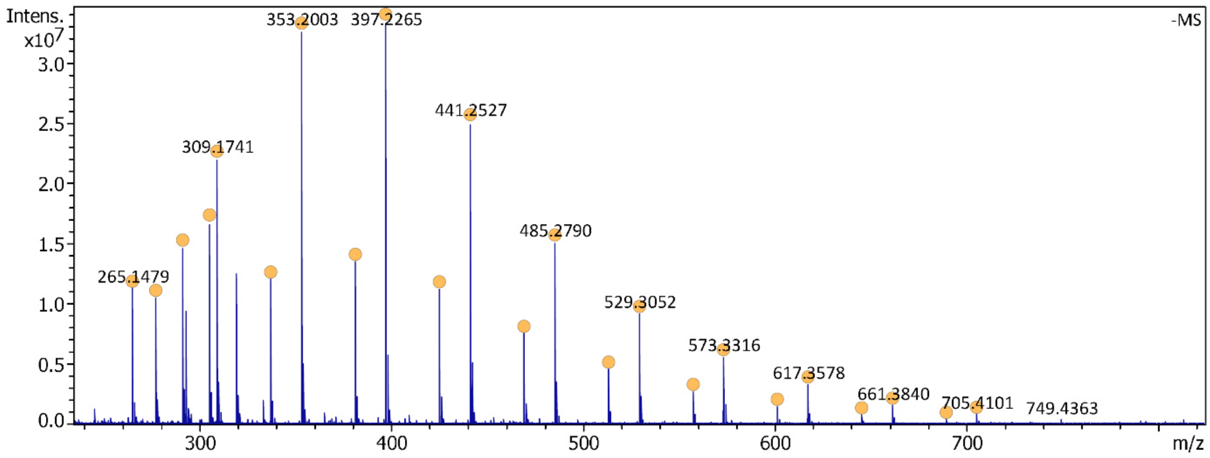

Meas. m/z	Ion Formula	m/z	err [ppm]	mSigma	rdb	e⁻ Conf	N-Rule
265.1479	$C_{12}H_{25}O_4S$	265.1479	0.0	13.5	0.5	even	ok
277.1843	$C_{14}H_{29}O_3S$	277.1843	0.0	19.7	0.5	even	ok
291.2000	$C_{15}H_{31}O_3S$	291.1999	-0.0	14.4	0.5	even	ok
305.2156	$C_{16}H_{33}O_3S$	305.2156	-0.0	8.6	0.5	even	ok
309.1741	$C_{14}H_{29}O_5S$	309.1741	-0.0	3.9	0.5	even	ok
337.2054	$C_{16}H_{33}O_5S$	337.2054	-0.0	7.1	0.5	even	ok
353.2003	$C_{16}H_{33}O_6S$	353.2003	0.1	8.9	0.5	even	ok
381.2316	$C_{18}H_{37}O_6S$	381.2316	0.2	9.5	0.5	even	ok
397.2265	$C_{18}H_{37}O_7S$	397.2265	0.2	11.0	0.5	even	ok
425.2579	$C_{20}H_{41}O_7S$	425.2578	-0.1	10.0	0.5	even	ok
441.2527	$C_{20}H_{41}O_8S$	441.2528	0.1	6.6	0.5	even	ok
469.2840	$C_{22}H_{45}O_8S$	469.2841	0.0	11.7	0.5	even	ok
485.2790	$C_{22}H_{45}O_9S$	485.2790	0.0	8.8	0.5	even	ok
513.3103	$C_{24}H_{49}O_9S$	513.3103	-0.1	12.5	0.5	even	ok
529.3052	$C_{24}H_{49}O_{10}S$	529.3052	-0.1	10.7	0.5	even	ok
557.3366	$C_{26}H_{53}O_{10}S$	557.3365	-0.2	11.7	0.5	even	ok
573.3316	$C_{26}H_{53}O_{11}S$	573.3314	-0.2	14.6	0.5	even	ok
601.3629	$C_{28}H_{57}O_{11}S$	601.3627	-0.3	35.7	0.5	even	ok
617.3578	$C_{28}H_{57}O_{12}S$	617.3576	-0.2	24.1	0.5	even	ok
645.3892	$C_{30}H_{61}O_{12}S$	645.3889	-0.4	41.3	0.5	even	ok
661.3840	$C_{30}H_{61}O_{13}S$	661.3838	-0.2	24.4	0.5	even	ok
689.4153	$C_{32}H_{65}O_{13}S$	689.4151	-0.2	48.0	2.0	even	ok
705.4101	$C_{32}H_{65}O_{14}S$	705.4101	-0.1	43.7	2.0	even	ok

Fig. S6. Negative-ion APFD tandem mass spectra of the ion at *m/z* 397.2, in this case selected from dusy women Duschgel (cf. Figs S21, S22). APFD conditions: 1–2 µl of a solution at 5 µl ml^{-1} in methanol : water = 9 : 1, acquisition 16 × 1.5 s, desolvation gas 1.2 l min^{-1} at 140 °C, shield 3.8 kV, cap 4.3 kV. **a)** Partial spectrum in MS mode, **b)** precursor ion *m/z* 397.2 selected at a collision offset voltage of just 1 V (blue diamond marks the precursor ion), **c)** at collision offset voltage of 10 V, **d)** collision offset voltage at 20 V. The precursor ion intensities are provided in the spectra. As long as fragmentation does not play a role, these remain at about 1 × 10^{7} counts while a drop to 6.0 × 10^{5} occurs at 20 V. Even though, no fragment ion peaks do occur within the accessible *m/z* range. The signals at odd *m/z* values of 162.83, 184.36, 212.45, 250.64, 295.41, and 442.62 are noise peaks that are always present due to the age of the instrument. Their intensities essentially are constant throughout the spectra, e.g., *m/z* 212.45 appears at about 2.5 × 10^{6} counts.

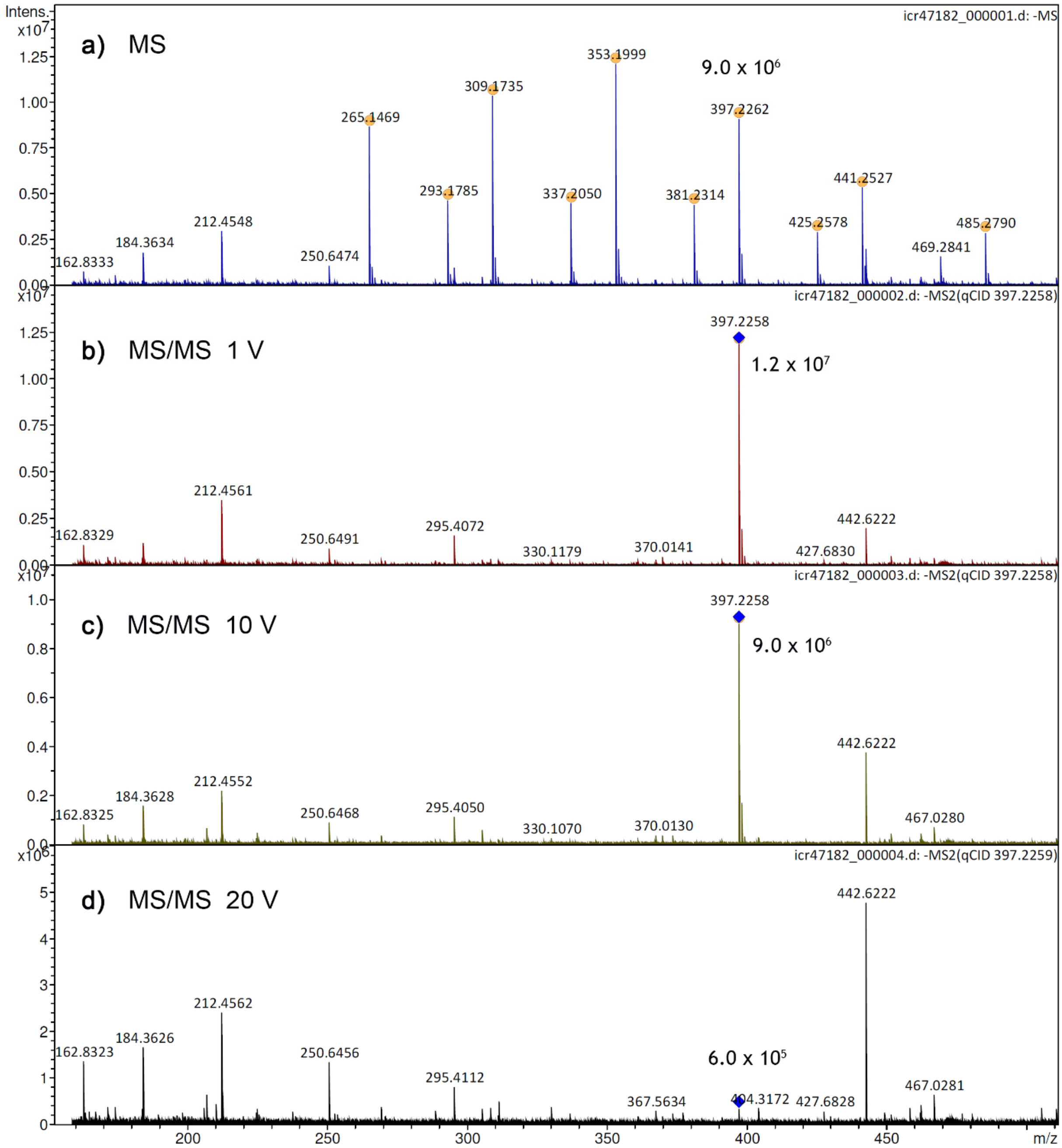

Fig. S7. Negative-ion APFD spectrum of Fairy ultra dishwashing liquid. APFD conditions: 1–2 µl of a solution at 3 µl ml^{-1} in methanol : water = 9 : 1, acquisition 16 × 2.0 s, desolvation gas 1.2 l min^{-1} at 140 °C, shield 4.0 kV, cap 4.5 kV. Yellow dots at the peak tops mark peaks with formula assignments in the list below the spectral plot.

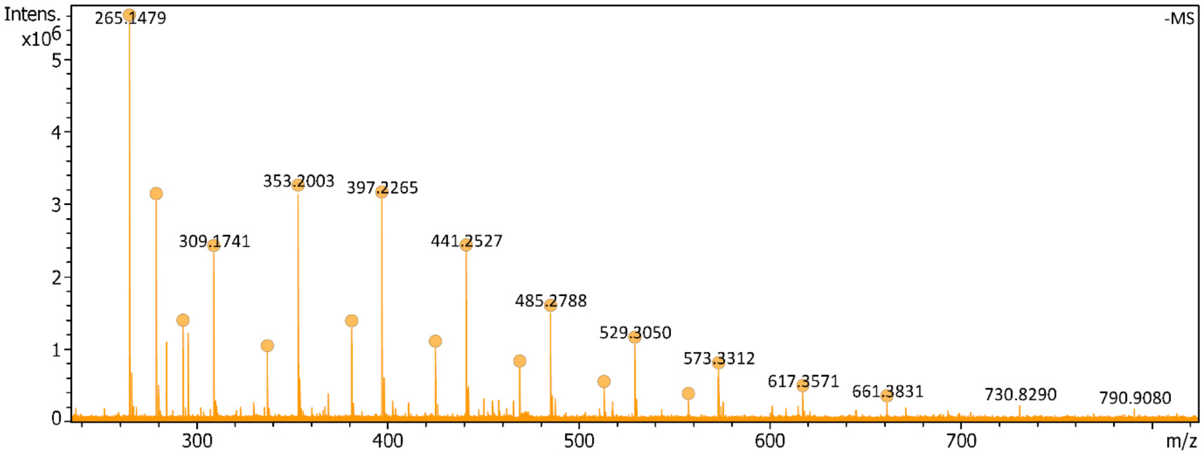

Meas. m/z	Ion Formula	m/z	err [ppm]	mSigma	rdb	e⁻ Conf	N-Rule
265.1479	C12H25O4S	265.1479	-0.1	7.1	0.5	even	ok
279.1636	C13H27O4S	279.1636	-0.1	14.1	0.5	even	ok
293.1793	C14H29O4S	293.1792	-0.2	19.5	0.5	even	ok
309.1741	C14H29O5S	309.1741	-0.1	13.6	0.5	even	ok
337.2054	C16H33O5S	337.2054	0.0	21.4	0.5	even	ok
353.2003	C16H33O6S	353.2003	-0.0	14.7	0.5	even	ok
381.2316	C18H37O6S	381.2316	0.2	18.0	0.5	even	ok
397.2265	C18H37O7S	397.2265	0.1	5.8	0.5	even	ok
425.2579	C20H41O7S	425.2578	-0.0	25.6	0.5	even	ok
441.2527	C20H41O8S	441.2528	0.2	18.3	0.5	even	ok
469.2839	C22H45O8S	469.2841	0.3	89.3	0.5	even	ok
485.2788	C22H45O9S	485.2790	0.4	24.3	0.5	even	ok
513.3104	C24H49O9S	513.3103	-0.3	33.6	0.5	even	ok
529.3050	C24H49O10S	529.3052	0.3	34.3	0.5	even	ok
557.3360	C26H53O10S	557.3365	0.8	38.6	0.5	even	ok
573.3312	C26H53O11S	573.3314	0.4	33.2	0.5	even	ok
617.3571	C28H57O12S	617.3576	0.9	46.2	2.0	even	ok
661.3831	C30H61O13S	661.3838	1.2	44.8	2.0	even	ok

Fig. S8. Negative-ion ESI spectrum of Fairy ultra dishwashing liquid. ESI conditions: 6 µl min^{-1} of a solution at 0.1 µl ml^{-1} in methanol : water = 9 : 1. Yellow dots at the peak tops mark peaks with formula assignments in the list below the spectral plot.

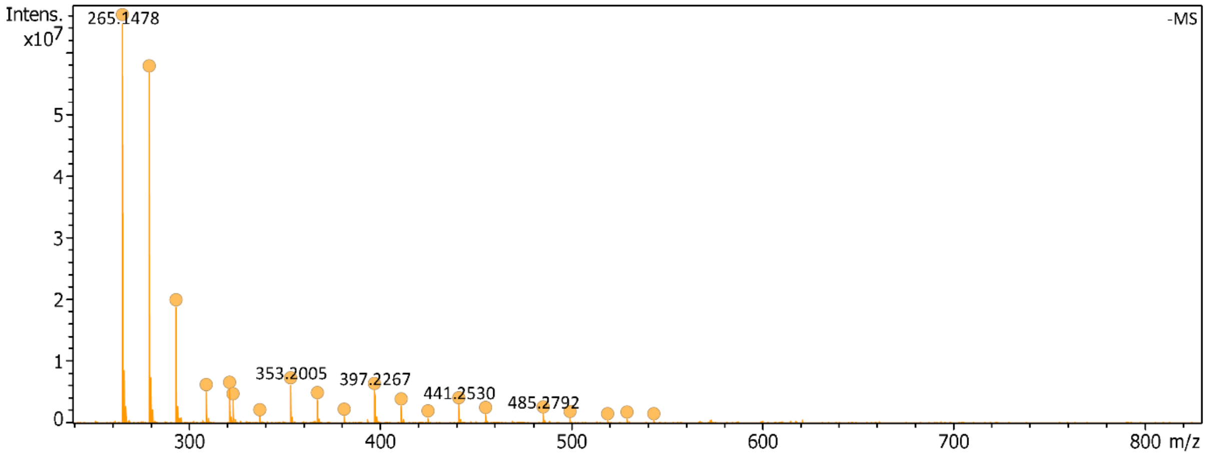

Meas. m/z	Ion Formula	m/z	err [ppm]	mSigma	rdb	e⁻ Conf	N-Rule
265.1478	C12H25O4S	265.1479	0.4	3.4	2.0	even	ok
279.1634	C13H27O4S	279.1636	0.7	6.3	2.0	even	ok
293.1792	C14H29O4S	293.1792	-0.1	5.7	2.0	even	ok
309.1742	C14H29O5S	309.1741	-0.3	8.7	2.0	even	ok
321.2106	C16H33O4S	321.2105	-0.4	10.3	2.0	even	ok
323.1899	C15H31O5S	323.1898	-0.3	10.6	2.0	even	ok
337.2056	C16H33O5S	337.2054	-0.4	27.4	2.0	even	ok
353.2005	C16H33O6S	353.2003	-0.4	9.1	2.0	even	ok
367.2162	C17H35O6S	367.2160	-0.5	5.3	2.0	even	ok
381.2318	C18H37O6S	381.2316	-0.4	31.0	2.0	even	ok
397.2267	C18H37O7S	397.2265	-0.4	15.2	2.0	even	ok
411.2424	C19H39O7S	411.2422	-0.5	24.5	2.0	even	ok
425.2581	C20H41O7S	425.2578	-0.5	28.9	2.0	even	ok
441.2530	C20H41O8S	441.2528	-0.5	21.3	2.0	even	ok
455.2686	C21H43O8S	455.2684	-0.5	47.3	2.0	even	ok
485.2792	C22H45O9S	485.2790	-0.4	30.3	2.0	even	ok
499.2950	C23H47O9S	499.2946	-0.7	36.0	2.0	even	ok
519.0190	C29H11O8S	519.0180	-2.0	n.a.	26.0	even	ok
529.3054	C24H49O10S	529.3052	-0.4	33.1	2.0	even	ok
543.3213	C25H51O10S	543.3208	-0.9	177.3	2.0	even	ok

Fig. S9. Negative-ion APFD spectrum of Fairy ultra plus dishwashing liquid. APFD conditions: 1–2 µl of a solution at 3 µl ml^{-1} in methanol : water = 9 : 1, acquisition 16 × 1.5 s, desolvation gas 1.2 l min^{-1} at 140 °C, shield 3.5 kV, cap 4.0 kV. Yellow dots at the peak tops mark peaks with formula assignments in the list below the spectral plot.

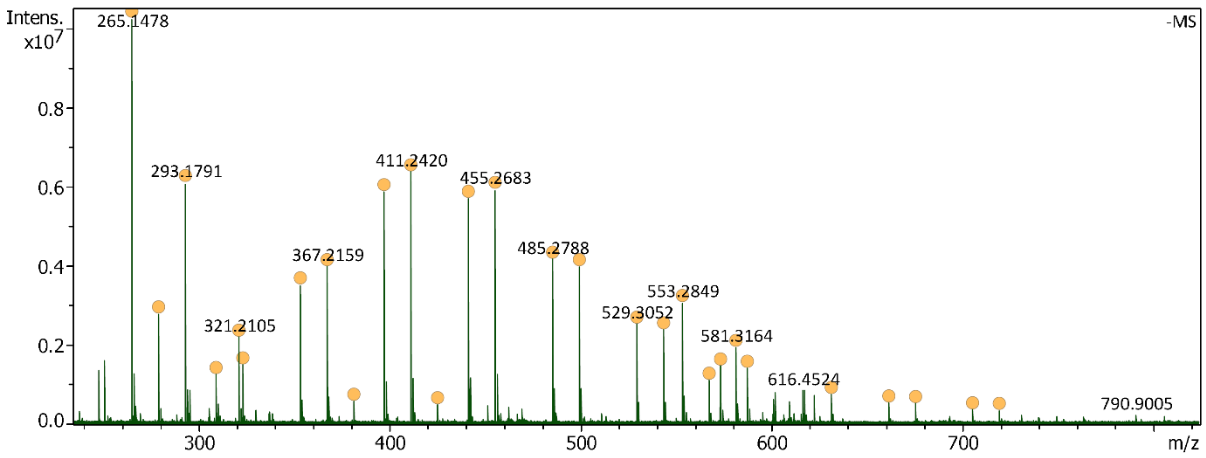

Meas. m/z	Ion Formula	m/z	err [ppm]	mSigma	rdb	e⁻ Conf	N-Rule
265.1478	C12H25O4S	265.1479	0.2	6.4	0.5	even	ok
279.1635	C13H27O4S	279.1636	0.1	7.8	0.5	even	ok
293.1791	C14H29O4S	293.1792	0.2	7.5	0.5	even	ok
309.1742	C14H29O5S	309.1741	-0.2	17.0	0.5	even	ok
321.2105	C16H33O4S	321.2105	-0.1	11.6	0.5	even	ok
323.1898	C15H31O5S	323.1898	-0.1	24.0	0.5	even	ok
353.2003	C16H33O6S	353.2003	0.0	5.9	0.5	even	ok
367.2159	C17H35O6S	367.2160	0.1	15.6	0.5	even	ok
381.2317	C18H37O6S	381.2316	-0.2	28.5	0.5	even	ok
397.2264	C18H37O7S	397.2265	0.4	10.7	0.5	even	ok
411.2420	C19H39O7S	411.2422	0.4	16.0	0.5	even	ok
425.2579	C20H41O7S	425.2578	-0.2	28.9	0.5	even	ok
441.2526	C20H41O8S	441.2528	0.3	15.7	0.5	even	ok
455.2683	C21H43O8S	455.2684	0.3	24.2	0.5	even	ok
485.2788	C22H45O9S	485.2790	0.3	28.2	0.5	even	ok
499.2945	C23H47O9S	499.2946	0.3	19.6	0.5	even	ok
529.3052	C24H49O10S	529.3052	0.1	40.9	0.5	even	ok
543.3208	C25H51O10S	543.3208	0.1	35.6	0.5	even	ok
553.2849	C29H45O8S	553.2841	-1.5	53.3	7.5	even	ok
567.3008	C30H47O8S	567.2997	-1.9	49.3	7.5	even	ok
573.3313	C26H53O11S	573.3314	0.1	39.9	0.5	even	ok
581.3164	C31H49O8S	581.3154	-1.8	48.4	7.5	even	ok
587.3471	C27H55O11S	587.3471	-0.1	38.0	0.5	even	ok
631.3734	C29H59O12S	631.3733	-0.1	38.2	0.5	even	ok
661.3840	C30H61O13S	661.3838	-0.2	75.9	0.5	even	ok
675.3996	C31H63O13S	675.3995	-0.2	78.8	0.5	even	ok
705.4107	C32H65O14S	705.4101	-0.9	78.9	0.5	even	ok
719.4262	C33H67O14S	719.4257	-0.7	75.4	0.5	even	ok

Fig. S10. Negative-ion ESI spectrum of Fairy ultra plus dishwashing liquid. ESI conditions: 6 µl min^{-1} of a solution at 0.1 µl ml^{-1} in methanol : water = 9 : 1. Yellow dots at the peak tops mark peaks with formula assignments in the list below the spectral plot.

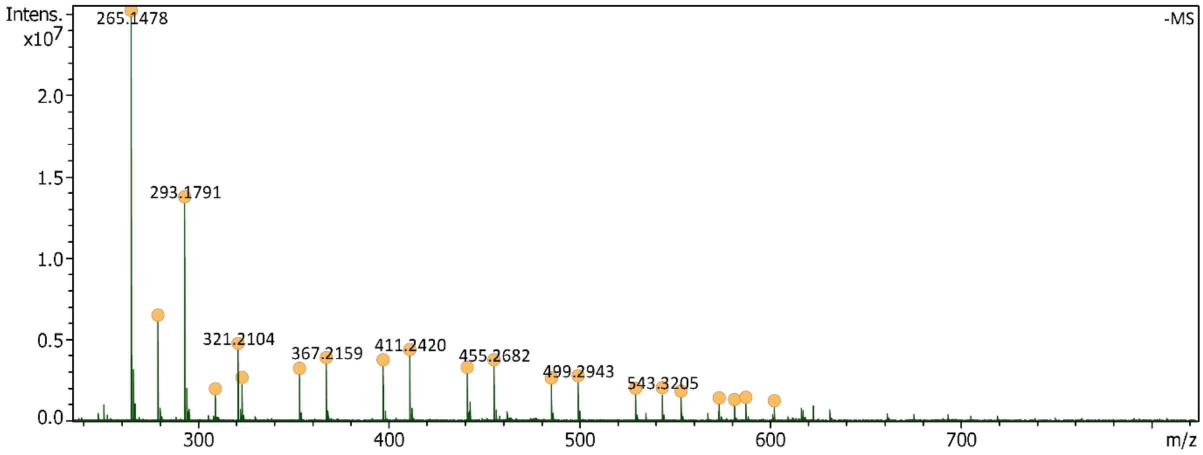

Meas. m/z	Ion Formula	m/z	err [ppm]	mSigma	rdb	e⁻ Conf	N-Rule
265.1478	C12H25O4S	265.1479	0.5	3.5	0.5	even	ok
279.1635	C13H27O4S	279.1636	0.2	6.8	0.5	even	ok
293.1791	C14H29O4S	293.1792	0.5	5.1	0.5	even	ok
309.1741	C14H29O5S	309.1741	0.0	11.2	0.5	even	ok
321.2104	C16H33O4S	321.2105	0.2	7.8	0.5	even	ok
323.1897	C15H31O5S	323.1898	0.1	8.6	0.5	even	ok
353.2003	C16H33O6S	353.2003	0.2	12.4	0.5	even	ok
367.2159	C17H35O6S	367.2160	0.3	5.5	0.5	even	ok
397.2264	C18H37O7S	397.2265	0.4	16.0	0.5	even	ok
411.2420	C19H39O7S	411.2422	0.4	16.0	0.5	even	ok
441.2526	C20H41O8S	441.2528	0.4	20.3	0.5	even	ok
455.2682	C21H43O8S	455.2684	0.4	44.7	0.5	even	ok
485.2787	C22H45O9S	485.2790	0.5	12.3	0.5	even	ok
499.2943	C23H47O9S	499.2946	0.6	24.2	0.5	even	ok
529.3049	C24H49O10S	529.3052	0.6	23.9	0.5	even	ok
543.3205	C25H51O10S	543.3208	0.7	40.2	0.5	even	ok
553.2848	C29H45O8S	553.2841	-1.3	49.9	7.5	even	ok
573.3310	C26H53O11S	573.3314	0.7	33.2	0.5	even	ok
581.3160	C31H49O8S	581.3154	-1.0	59.5	7.5	even	ok
587.3466	C27H55O11S	587.3471	0.7	36.6	0.5	even	ok
602.0547	C33H16NO9S	602.0551	0.8	n.a.	26.5	even	ok

Fig. S11. Negative-ion APFD spectrum of Robbyrob Scheibenfrostschutz liquid. APFD conditions: 1–2 µl of a solution at 25 µl ml^{-1} in methanol : water = 9 : 1, acquisition 16 × 1.5 s, desolvation gas 1.2 l min^{-1} at 140 °C, shield 3.5 kV, cap 4.0 kV. Yellow dots at the peak tops mark peaks with formula assignments in the list below the spectral plot.

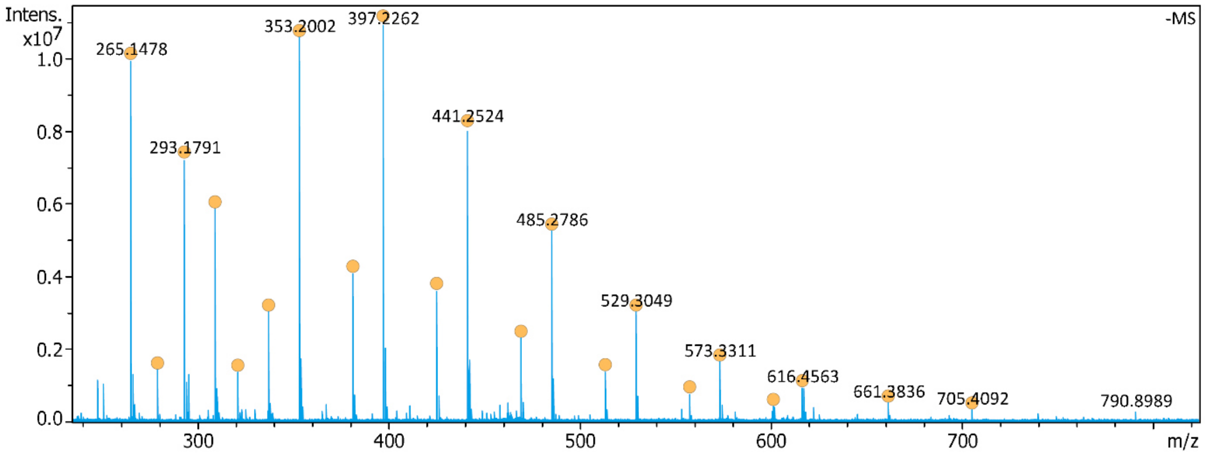

Meas. m/z	Ion Formula	m/z	err [ppm]	mSigma	rdb	e⁻ Conf	N-Rule
265.1478	C12H25O4S	265.1479	0.3	5.7	0.5	even	ok
279.1635	C13H27O4S	279.1636	0.0	20.2	0.5	even	ok
293.1791	C14H29O4S	293.1792	0.2	6.9	0.5	even	ok
309.1741	C14H29O5S	309.1741	0.1	6.9	0.5	even	ok
321.2105	C16H33O4S	321.2105	-0.1	23.7	0.5	even	ok
337.2054	C16H33O5S	337.2054	0.1	19.1	0.5	even	ok
353.2002	C16H33O6S	353.2003	0.5	7.5	0.5	even	ok
381.2316	C18H37O6S	381.2316	0.2	16.7	0.5	even	ok
397.2262	C18H37O7S	397.2265	0.9	11.7	0.5	even	ok
425.2577	C20H41O7S	425.2578	0.4	19.7	0.5	even	ok
441.2524	C20H41O8S	441.2528	0.7	14.9	0.5	even	ok
469.2839	C22H45O8S	469.2841	0.3	22.3	0.5	even	ok
485.2786	C22H45O9S	485.2790	0.8	17.0	0.5	even	ok
513.3100	C24H49O9S	513.3103	0.5	22.7	0.5	even	ok
529.3049	C24H49O10S	529.3052	0.5	31.1	0.5	even	ok
557.3364	C26H53O10S	557.3365	0.2	39.8	0.5	even	ok
573.3311	C26H53O11S	573.3314	0.5	33.6	0.5	even	ok
601.3624	C28H57O11S	601.3627	0.5	56.5	0.5	even	ok
616.4563	C41H62NOS	616.4558	-0.9	195.4	11.5	even	ok
661.3836	C30H61O13S	661.3838	0.3	40.1	2.0	even	ok
705.4092	C32H65O14S	705.4101	1.2	91.7	2.0	even	ok

Fig. S12. Negative-ion ESI spectrum of Robbyrob Scheibenfrostschutz liquid. ESI conditions: 6 µl min^{-1} of a solution at 0.5 µl ml^{-1} in methanol : water = 9 : 1. Yellow dots at the peak tops mark peaks with formula assignments in the list below the spectral plot.

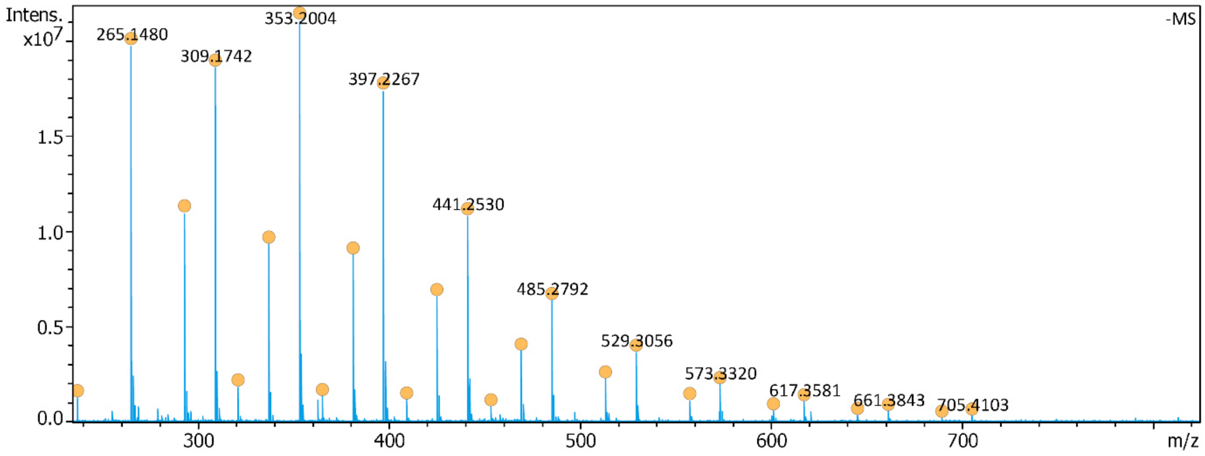

Meas. m/z	Ion Formula	m/z	err [ppm]	mSigma	rdb	e⁻ Conf	N-Rule
237.1167	C10H21O4S	237.1166	-0.6	21.5	0.5	even	ok
265.1480	C12H25O4S	265.1479	-0.3	4.5	0.5	even	ok
293.1794	C14H29O4S	293.1792	-0.5	4.2	2.0	even	ok
309.1742	C14H29O5S	309.1741	-0.2	6.3	2.0	even	ok
321.2108	C16H33O4S	321.2105	-0.9	18.8	0.5	even	ok
337.2056	C16H33O5S	337.2054	-0.6	7.4	2.0	even	ok
353.2004	C16H33O6S	353.2003	-0.3	8.0	2.0	even	ok
365.2371	C18H37O5S	365.2367	-0.9	26.2	2.0	even	ok
381.2318	C18H37O6S	381.2316	-0.5	7.3	2.0	even	ok
397.2267	C18H37O7S	397.2265	-0.5	12.9	2.0	even	ok
409.2633	C20H41O6S	409.2629	-0.9	27.9	2.0	even	ok
425.2582	C20H41O7S	425.2578	-0.7	13.2	2.0	even	ok
441.2530	C20H41O8S	441.2528	-0.6	14.0	2.0	even	ok
453.2897	C22H45O7S	453.2891	-1.1	32.0	2.0	even	ok
469.2844	C22H45O8S	469.2841	-0.8	22.1	2.0	even	ok
485.2792	C22H45O9S	485.2790	-0.5	19.1	2.0	even	ok
513.3108	C24H49O9S	513.3103	-0.9	34.4	2.0	even	ok
529.3056	C24H49O10S	529.3052	-0.8	30.4	2.0	even	ok
557.3369	C26H53O10S	557.3365	-0.8	31.8	2.0	even	ok
573.3320	C26H53O11S	573.3314	-1.0	33.7	2.0	even	ok
601.3634	C28H57O11S	601.3627	-1.1	37.7	2.0	even	ok
617.3581	C28H57O12S	617.3576	-0.8	41.1	2.0	even	ok
645.3896	C30H61O12S	645.3889	-1.0	62.6	2.0	even	ok
661.3843	C30H61O13S	661.3838	-0.6	66.3	2.0	even	ok
689.4155	C32H65O13S	689.4151	-0.5	287.9	2.0	even	ok
705.4103	C32H65O14S	705.4101	-0.3	73.1	2.0	even	ok

Fig. S13. Negative-ion APFD spectrum of Today Seife Aqua. APFD conditions: 1–2 µl of a solution at 5 µl ml^{-1} in methanol : water = 9 : 1, acquisition 16 × 2.0 s, desolvation gas 1.2 l min^{-1} at 140 °C, shield 3.5 kV, cap 4.0 kV. Yellow dots at the peak tops mark peaks with formula assignments in the list below the spectral plot.

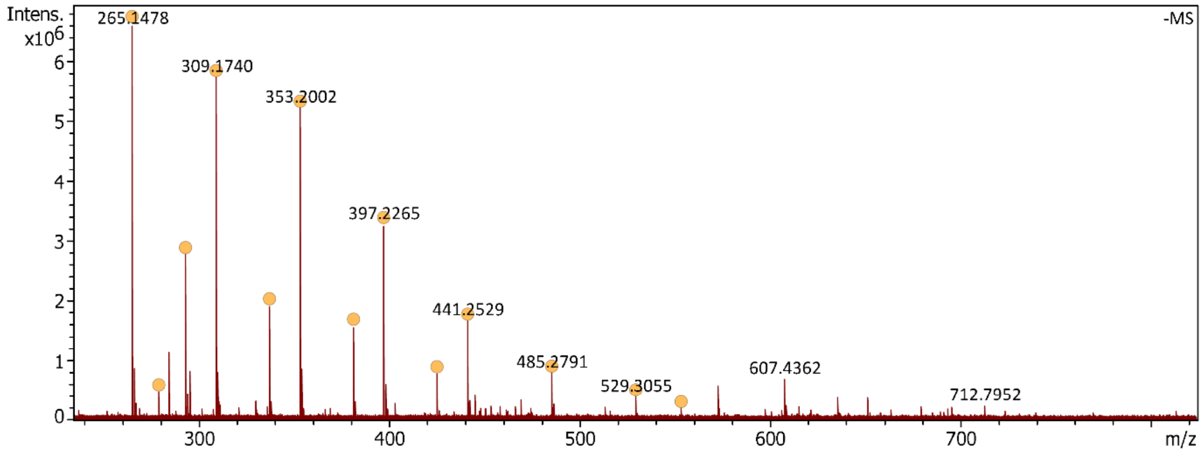

Meas. m/z	Ion Formula	m/z	err [ppm]	mSigma	rdb	e⁻ Conf	N-Rule
265.1478	C12H25O4S	265.1479	0.5	6.0	0.5	even	ok
279.1636	C13H27O4S	279.1636	-0.1	n.a.	0.5	even	ok
293.1792	C14H29O4S	293.1792	0.1	8.0	0.5	even	ok
309.1740	C14H29O5S	309.1741	0.5	7.7	0.5	even	ok
337.2055	C16H33O5S	337.2054	-0.2	21.7	0.5	even	ok
353.2002	C16H33O6S	353.2003	0.4	8.3	0.5	even	ok
381.2317	C18H37O6S	381.2316	-0.2	25.0	0.5	even	ok
397.2265	C18H37O7S	397.2265	0.2	17.4	0.5	even	ok
425.2579	C20H41O7S	425.2578	-0.2	27.9	0.5	even	ok
441.2529	C20H41O8S	441.2528	-0.3	30.9	0.5	even	ok
485.2791	C22H45O9S	485.2790	-0.3	32.8	0.5	even	ok
529.3055	C24H49O10S	529.3052	-0.6	34.6	0.5	even	ok
553.2846	C29H45O8S	553.2841	-1.0	n.a.	7.5	even	ok

Fig. S14. (*Top*) Negative-ion ESI spectrum of Today Seife Aqua. ESI conditions: 6 µl min^{-1} at 0.1 µl ml^{-1} in methanol : water = 9 : 1 (ApexQe). (*Bottom*) Tandem mass spectra (all at 25 V collision offset) of several precursor ions ranging from *m/z* 265 to *m/z* 441 (timsTOFflex).

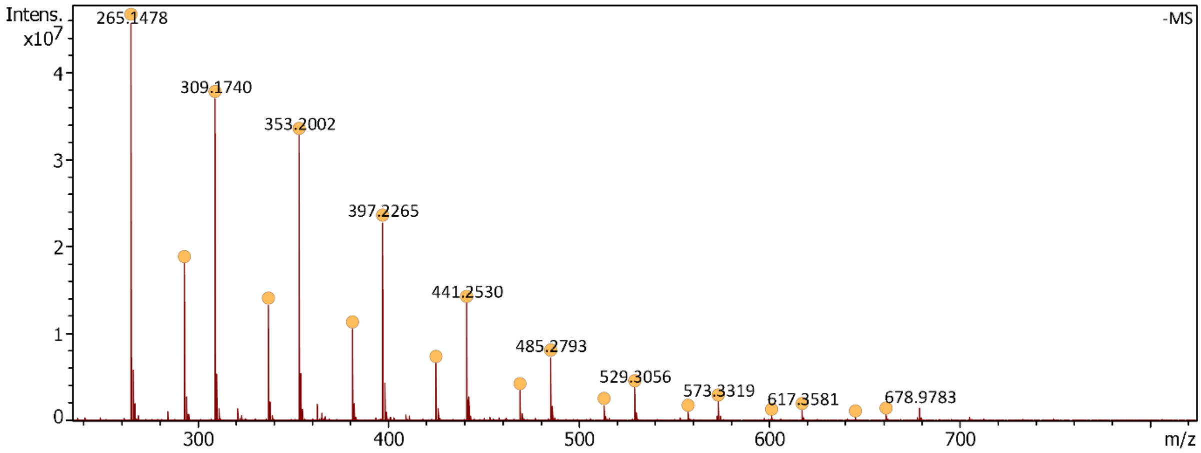

Meas. m/z	Ion Formula	m/z	err [ppm]	mSigma	rdb	e⁻ Conf	N-Rule
265.1478	C12H25O4S	265.1479	0.5	3.9	2.0	even	ok
293.1792	C14H29O4S	293.1792	0.0	4.8	2.0	even	ok
309.1740	C14H29O5S	309.1741	0.4	6.6	2.0	even	ok
337.2055	C16H33O5S	337.2054	-0.3	8.0	2.0	even	ok
353.2002	C16H33O6S	353.2003	0.3	9.4	2.0	even	ok
381.2318	C18H37O6S	381.2316	-0.4	11.2	2.0	even	ok
397.2265	C18H37O7S	397.2265	0.0	11.9	2.0	even	ok
425.2580	C20H41O7S	425.2578	-0.5	14.9	2.0	even	ok
441.2530	C20H41O8S	441.2528	-0.5	15.8	2.0	even	ok
469.2844	C22H45O8S	469.2841	-0.7	27.2	2.0	even	ok
485.2793	C22H45O9S	485.2790	-0.6	16.0	2.0	even	ok
513.3108	C24H49O9S	513.3103	-1.0	26.2	2.0	even	ok
529.3056	C24H49O10S	529.3052	-0.8	18.2	2.0	even	ok
557.3371	C26H53O10S	557.3365	-1.2	48.8	2.0	even	ok
573.3319	C26H53O11S	573.3314	-0.9	33.7	2.0	even	ok
601.3633	C28H57O11S	601.3627	-1.0	37.2	2.0	even	ok
617.3581	C28H57O12S	617.3576	-0.8	37.2	2.0	even	ok
645.3896	C30H61O12S	645.3889	-1.0	44.8	2.0	even	ok
661.3846	C30H61O13S	661.3838	-1.2	66.6	2.0	even	ok

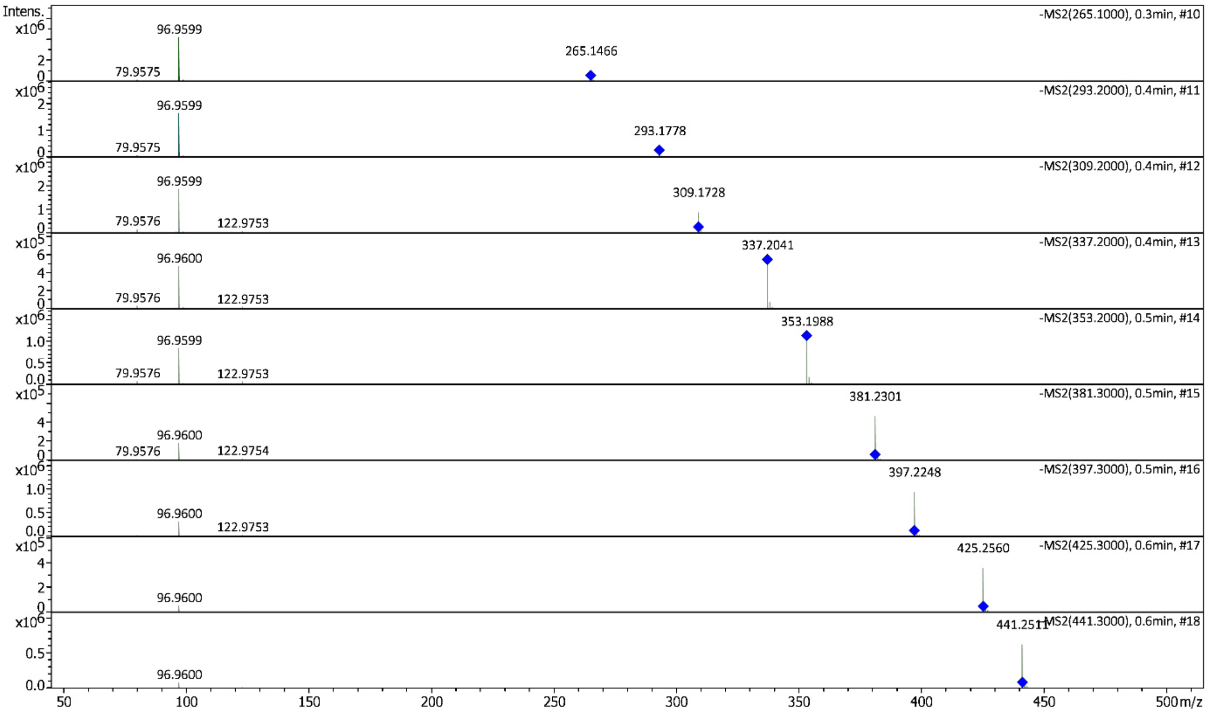

Fig. S15. Negative-ion APFD spectrum of Today Seife sensitive. APFD conditions: 1–2 µl of a solution at 5 µl ml^{-1} in methanol : water = 9 : 1, acquisition 16 × 1.5 s, desolvation gas 1.2 l min^{-1} at 140 °C, shield 3.5 kV, cap 4.0 kV. Yellow dots at the peak tops mark peaks with formula assignments in the list below the spectral plot.

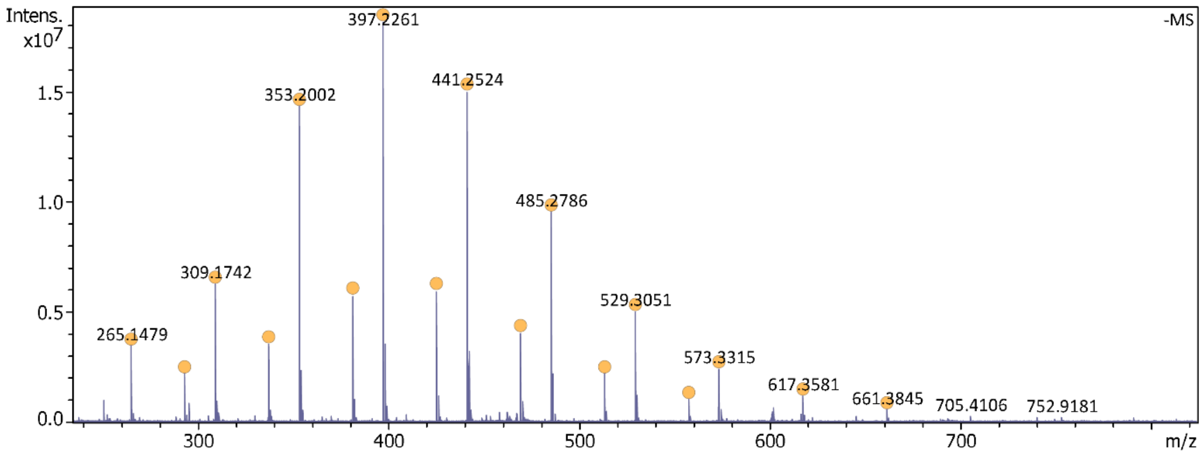

Meas. m/z	Ion Formula	m/z	err [ppm]	mSigma	rdb	e⁻ Conf	N-Rule
265.1479	C12H25O4S	265.1479	-0.2	7.2	0.5	even	ok
293.1793	C14H29O4S	293.1792	-0.4	19.5	0.5	even	ok
309.1742	C14H29O5S	309.1741	-0.2	7.7	0.5	even	ok
337.2055	C16H33O5S	337.2054	-0.3	8.4	0.5	even	ok
353.2002	C16H33O6S	353.2003	0.4	8.2	0.5	even	ok
381.2317	C18H37O6S	381.2316	-0.1	9.2	0.5	even	ok
397.2261	C18H37O7S	397.2265	1.0	10.6	0.5	even	ok
425.2578	C20H41O7S	425.2578	0.1	16.4	0.5	even	ok
441.2524	C20H41O8S	441.2528	0.9	15.5	0.5	even	ok
469.2842	C22H45O8S	469.2841	-0.2	17.0	0.5	even	ok
485.2786	C22H45O9S	485.2790	0.7	17.4	0.5	even	ok
513.3105	C24H49O9S	513.3103	-0.5	31.5	0.5	even	ok
529.3051	C24H49O10S	529.3052	0.1	28.9	0.5	even	ok
557.3369	C26H53O10S	557.3365	-0.8	36.8	0.5	even	ok
573.3315	C26H53O11S	573.3314	-0.1	29.5	0.5	even	ok
617.3581	C28H57O12S	617.3576	-0.8	38.0	0.5	even	ok
661.3845	C30H61O13S	661.3838	-1.0	40.0	0.5	even	ok

Fig. S16. Negative-ion ESI spectrum of Today Seife sensitive. ESI conditions: 6 µl min^{-1} of a solution at 0.1 µl ml^{-1} in methanol : water = 9 : 1. Yellow dots at the peak tops mark peaks with formula assignments in the list below the spectral plot.

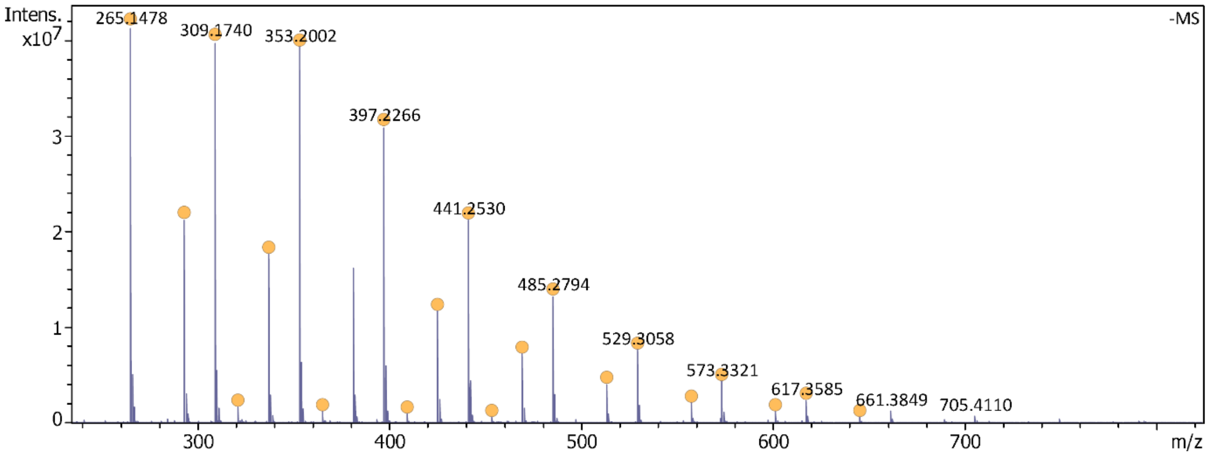

Meas. m/z	Ion Formula	m/z	err [ppm]	mSigma	rdb	e⁻ Conf	N-Rule
265.1478	C12H25O4S	265.1479	0.3	4.7	0.5	even	ok
293.1793	C14H29O4S	293.1792	-0.2	6.1	0.5	even	ok
309.1740	C14H29O5S	309.1741	0.2	8.1	0.5	even	ok
321.2107	C16H33O4S	321.2105	-0.7	10.8	0.5	even	ok
337.2056	C16H33O5S	337.2054	-0.5	6.9	0.5	even	ok
353.2002	C16H33O6S	353.2003	0.2	11.5	0.5	even	ok
365.2370	C18H37O5S	365.2367	-0.8	25.6	0.5	even	ok
397.2266	C18H37O7S	397.2265	-0.0	11.6	0.5	even	ok
409.2633	C20H41O6S	409.2629	-0.8	26.6	0.5	even	ok
425.2581	C20H41O7S	425.2578	-0.5	15.3	0.5	even	ok
441.2530	C20H41O8S	441.2528	-0.5	15.0	0.5	even	ok
453.2897	C22H45O7S	453.2891	-1.2	32.4	0.5	even	ok
469.2845	C22H45O8S	469.2841	-1.0	20.1	0.5	even	ok
485.2794	C22H45O9S	485.2790	-0.8	17.8	0.5	even	ok
513.3110	C24H49O9S	513.3103	-1.3	20.1	0.5	even	ok
529.3058	C24H49O10S	529.3052	-1.1	19.2	0.5	even	ok
557.3372	C26H53O10S	557.3365	-1.3	26.0	0.5	even	ok
573.3321	C26H53O11S	573.3314	-1.3	18.4	0.5	even	ok
601.3638	C28H57O11S	601.3627	-1.8	57.3	0.5	even	ok
617.3585	C28H57O12S	617.3576	-1.5	22.8	0.5	even	ok
645.3900	C30H61O12S	645.3889	-1.6	44.1	0.5	even	ok

Fig. S17. Negative-ion APFD spectrum of Balea milde Seife. APFD conditions: 1–2 µl of a solution at 5 µl ml^{-1} in methanol : water = 9 : 1, acquisition 16 × 1.5 s, desolvation gas 1.2 l min^{-1} at 140 °C, shield 3.5 kV, cap 4.0 kV. Yellow dots at the peak tops mark peaks with formula assignments in the list below the spectral plot.

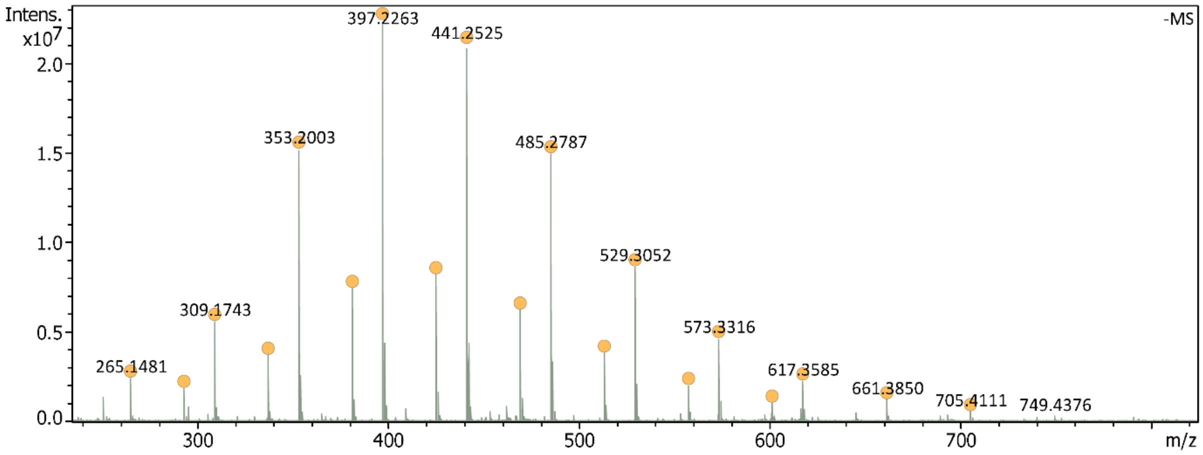

Meas. m/z	Ion Formula	m/z	err [ppm]	mSigma	rdb	e⁻ Conf	N-Rule
265.1481	C12H25O4S	265.1479	-0.7	7.2	0.5	even	ok
293.1794	C14H29O4S	293.1792	-0.8	8.2	0.5	even	ok
309.1743	C14H29O5S	309.1741	-0.7	8.1	0.5	even	ok
337.2057	C16H33O5S	337.2054	-0.8	9.8	0.5	even	ok
353.2003	C16H33O6S	353.2003	0.0	8.7	0.5	even	ok
381.2318	C18H37O6S	381.2316	-0.4	16.6	0.5	even	ok
397.2263	C18H37O7S	397.2265	0.7	11.1	0.5	even	ok
425.2580	C20H41O7S	425.2578	-0.3	12.8	0.5	even	ok
441.2525	C20H41O8S	441.2528	0.6	16.9	0.5	even	ok
469.2843	C22H45O8S	469.2841	-0.5	28.8	0.5	even	ok
485.2787	C22H45O9S	485.2790	0.6	18.4	0.5	even	ok
513.3107	C24H49O9S	513.3103	-0.8	18.3	0.5	even	ok
529.3052	C24H49O10S	529.3052	-0.1	21.4	0.5	even	ok
557.3371	C26H53O10S	557.3365	-1.0	25.6	0.5	even	ok
573.3316	C26H53O11S	573.3314	-0.4	33.9	0.5	even	ok
601.3637	C28H57O11S	601.3627	-1.7	35.7	0.5	even	ok
617.3585	C28H57O12S	617.3576	-1.5	21.2	0.5	even	ok
661.3850	C30H61O13S	661.3838	-1.7	49.3	0.5	even	ok
705.4111	C32H65O14S	705.4101	-1.5	72.5	2.0	even	ok

Fig. S18. Negative-ion ESI spectrum of Balea milde Seife. ESI conditions: 6 µl min^{-1} of a solution at 0.1 µl ml^{-1} in methanol : water = 9 : 1. Yellow dots at the peak tops mark peaks with formula assignments in the list below the spectral plot.

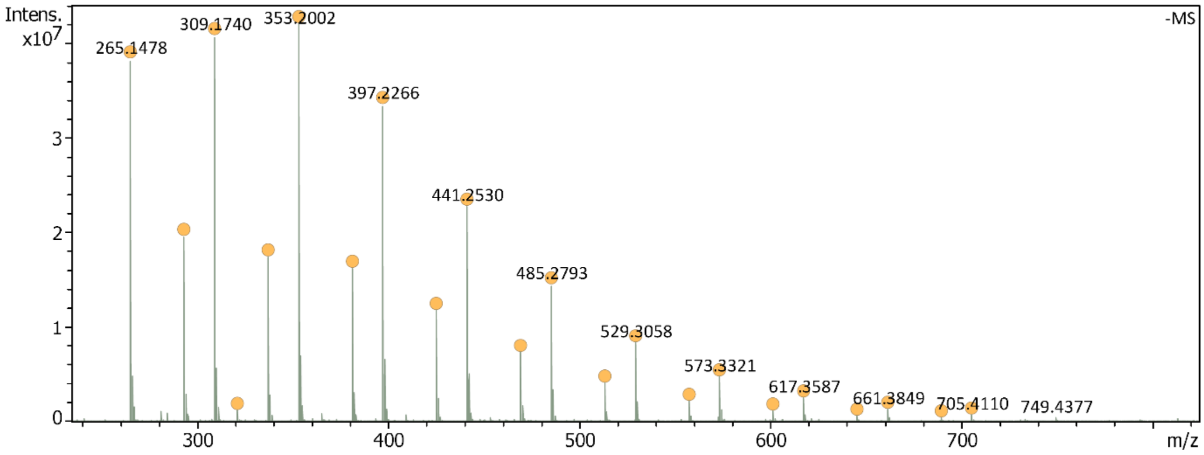

Meas. m/z	Ion Formula	m/z	err [ppm]	mSigma	rdb	e⁻ Conf	N-Rule
265.1478	C12H25O4S	265.1479	0.3	4.0	0.5	even	ok
293.1793	C14H29O4S	293.1792	-0.3	4.8	0.5	even	ok
309.1740	C14H29O5S	309.1741	0.2	8.5	0.5	even	ok
321.2108	C16H33O4S	321.2105	-0.8	21.4	2.0	even	ok
337.2056	C16H33O5S	337.2054	-0.5	6.7	0.5	even	ok
353.2002	C16H33O6S	353.2003	0.3	10.1	0.5	even	ok
381.2318	C18H37O6S	381.2316	-0.4	8.6	0.5	even	ok
397.2266	C18H37O7S	397.2265	-0.0	10.9	0.5	even	ok
425.2581	C20H41O7S	425.2578	-0.5	12.9	0.5	even	ok
441.2530	C20H41O8S	441.2528	-0.5	12.7	0.5	even	ok
469.2845	C22H45O8S	469.2841	-0.9	16.0	0.5	even	ok
485.2793	C22H45O9S	485.2790	-0.7	15.2	0.5	even	ok
513.3110	C24H49O9S	513.3103	-1.4	27.8	0.5	even	ok
529.3058	C24H49O10S	529.3052	-1.1	19.4	0.5	even	ok
557.3373	C26H53O10S	557.3365	-1.4	22.2	0.5	even	ok
573.3321	C26H53O11S	573.3314	-1.2	19.2	0.5	even	ok
601.3636	C28H57O11S	601.3627	-1.5	36.2	0.5	even	ok
617.3587	C28H57O12S	617.3576	-1.7	23.3	0.5	even	ok
645.3899	C30H61O12S	645.3889	-1.6	44.8	0.5	even	ok
661.3849	C30H61O13S	661.3838	-1.7	44.8	2.0	even	ok
689.4157	C32H65O13S	689.4151	-0.9	68.2	2.0	even	ok
705.4110	C32H65O14S	705.4101	-1.4	68.9	2.0	even	ok

Fig. S19. Negative-ion APFD spectrum of Ombia Duschgel. APFD conditions: 1–2 µl of a solution at 5 µl ml^{-1} in methanol : water = 9 : 1, acquisition 16 × 1.5 s, desolvation gas 1.2 l min^{-1} at 140 °C, shield 3.5 kV, cap 4.0 kV. Yellow dots at the peak tops mark peaks with formula assignments in the list below the spectral plot.

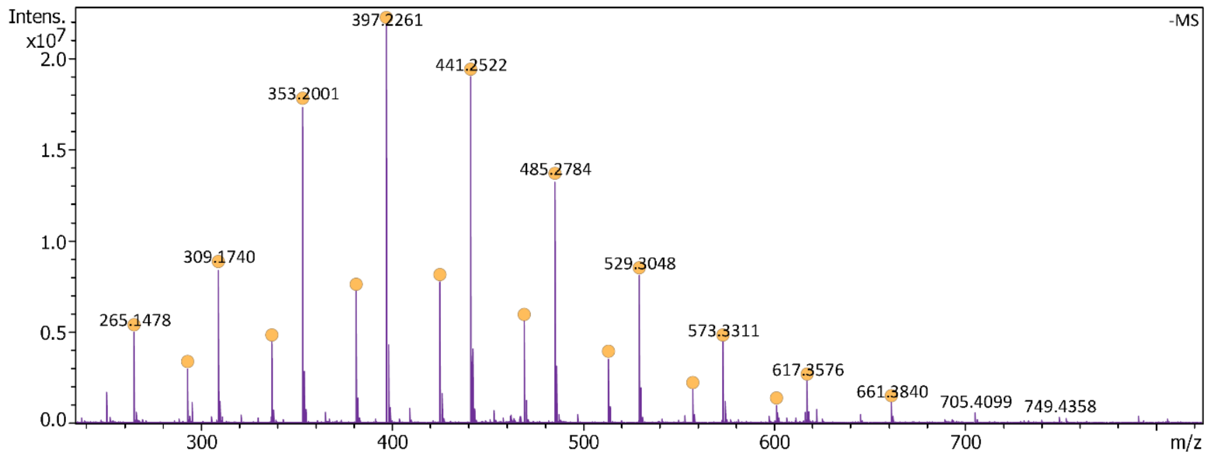

Meas. m/z	Ion Formula	m/z	err [ppm]	mSigma	rdb	e⁻ Conf	N-Rule
265.1478	C12H25O4S	265.1479	0.4	6.1	0.5	even	ok
293.1791	C14H29O4S	293.1792	0.2	8.3	0.5	even	ok
309.1740	C14H29O5S	309.1741	0.4	7.9	0.5	even	ok
337.2054	C16H33O5S	337.2054	0.2	5.1	0.5	even	ok
353.2001	C16H33O6S	353.2003	0.8	8.1	0.5	even	ok
381.2315	C18H37O6S	381.2316	0.4	6.3	0.5	even	ok
397.2261	C18H37O7S	397.2265	1.2	9.5	0.5	even	ok
425.2577	C20H41O7S	425.2578	0.4	9.1	0.5	even	ok
441.2522	C20H41O8S	441.2528	1.2	15.2	0.5	even	ok
469.2839	C22H45O8S	469.2841	0.4	14.3	0.5	even	ok
485.2784	C22H45O9S	485.2790	1.2	13.9	0.5	even	ok
513.3101	C24H49O9S	513.3103	0.3	14.2	0.5	even	ok
529.3048	C24H49O10S	529.3052	0.7	18.5	0.5	even	ok
557.3363	C26H53O10S	557.3365	0.3	36.3	0.5	even	ok
573.3311	C26H53O11S	573.3314	0.6	16.4	0.5	even	ok
601.3628	C28H57O11S	601.3627	-0.2	35.6	0.5	even	ok
617.3576	C28H57O12S	617.3576	0.1	34.2	0.5	even	ok
661.3840	C30H61O13S	661.3838	-0.2	20.6	0.5	even	ok

Fig. S20. Negative-ion ESI spectrum of Ombia Duschgel. ESI conditions: 6 µl min^{-1} of a solution at 0.1 µl ml^{-1} in methanol : water = 9 : 1. Yellow dots at the peak tops mark peaks with formula assignments in the list below the spectral plot.

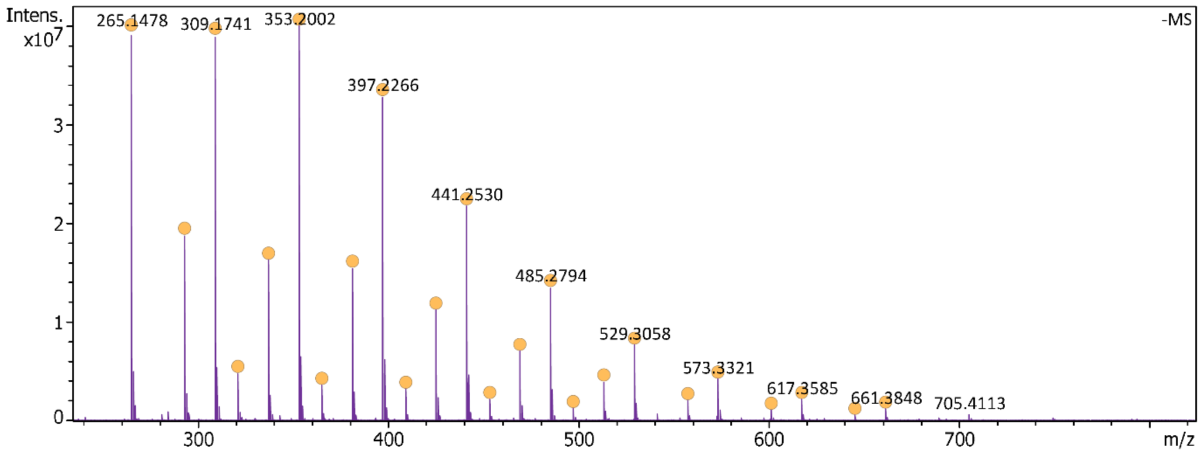

Meas. m/z	Ion Formula	m/z	err [ppm]	mSigma	rdb	e⁻ Conf	N-Rule
265.1478	C12H25O4S	265.1479	0.3	4.1	0.5	even	ok
293.1793	C14H29O4S	293.1792	-0.3	5.2	0.5	even	ok
309.1741	C14H29O5S	309.1741	0.2	8.3	0.5	even	ok
321.2107	C16H33O4S	321.2105	-0.7	11.8	0.5	even	ok
337.2056	C16H33O5S	337.2054	-0.5	9.6	0.5	even	ok
353.2002	C16H33O6S	353.2003	0.3	9.8	2.0	even	ok
365.2370	C18H37O5S	365.2367	-0.8	14.2	0.5	even	ok
381.2318	C18H37O6S	381.2316	-0.5	9.5	0.5	even	ok
397.2266	C18H37O7S	397.2265	-0.1	11.8	0.5	even	ok
409.2633	C20H41O6S	409.2629	-0.9	18.4	0.5	even	ok
425.2581	C20H41O7S	425.2578	-0.5	13.6	0.5	even	ok
441.2530	C20H41O8S	441.2528	-0.5	14.0	0.5	even	ok
453.2897	C22H45O7S	453.2891	-1.1	26.5	0.5	even	ok
469.2845	C22H45O8S	469.2841	-0.9	19.5	0.5	even	ok
485.2794	C22H45O9S	485.2790	-0.8	16.4	0.5	even	ok
497.3160	C24H49O8S	497.3154	-1.3	32.7	0.5	even	ok
513.3110	C24H49O9S	513.3103	-1.3	18.5	0.5	even	ok
529.3058	C24H49O10S	529.3052	-1.1	21.8	0.5	even	ok
557.3373	C26H53O10S	557.3365	-1.4	28.5	0.5	even	ok
573.3321	C26H53O11S	573.3314	-1.3	24.6	0.5	even	ok
601.3636	C28H57O11S	601.3627	-1.5	29.4	0.5	even	ok
617.3585	C28H57O12S	617.3576	-1.5	32.6	0.5	even	ok
645.3899	C30H61O12S	645.3889	-1.5	55.6	0.5	even	ok
661.3848	C30H61O13S	661.3838	-1.5	41.5	2.0	even	ok

Fig. S21. Negative-ion APFD spectrum of dusy women Duschgel. APFD conditions: 1–2 µl of a solution at 5 µl ml^{-1} in methanol : water = 9 : 1, acquisition 16 × 1.5 s, desolvation gas 1.2 l min^{-1} at 140 °C, shield 3.5 kV, cap 4.0 kV. Yellow dots at the peak tops mark peaks with formula assignments in the list below the spectral plot.

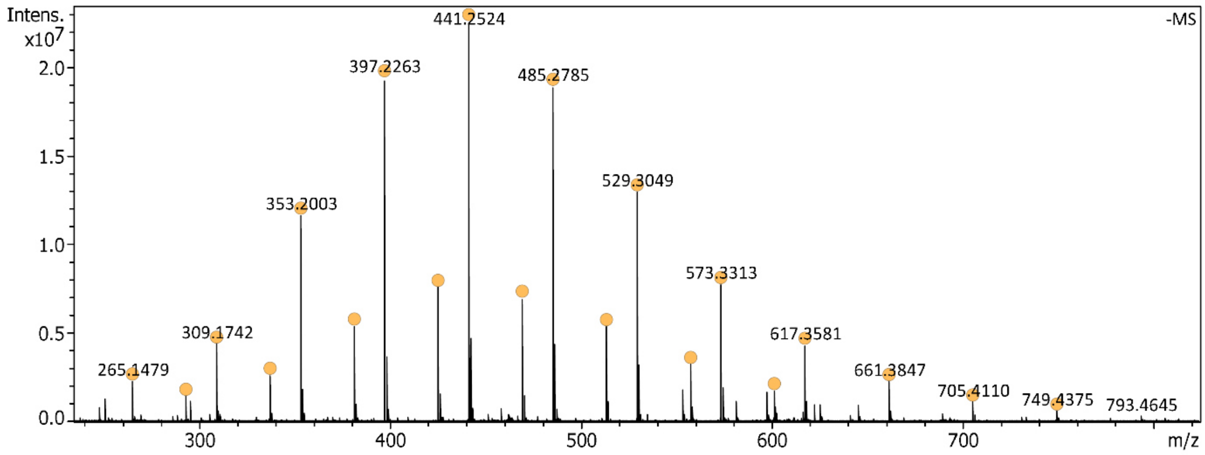

Meas. m/z	Ion Formula	m/z	err [ppm]	mSigma	rdb	e⁻ Conf	N-Rule
265.1479	C12H25O4S	265.1479	-0.1	6.5	0.5	even	ok
293.1793	C14H29O4S	293.1792	-0.3	23.7	0.5	even	ok
309.1742	C14H29O5S	309.1741	-0.2	8.5	0.5	even	ok
337.2055	C16H33O5S	337.2054	-0.4	11.7	0.5	even	ok
353.2003	C16H33O6S	353.2003	0.1	9.7	0.5	even	ok
381.2317	C18H37O6S	381.2316	-0.3	9.8	0.5	even	ok
397.2263	C18H37O7S	397.2265	0.6	11.3	0.5	even	ok
425.2580	C20H41O7S	425.2578	-0.3	15.9	0.5	even	ok
441.2524	C20H41O8S	441.2528	0.9	17.3	0.5	even	ok
469.2842	C22H45O8S	469.2841	-0.3	22.5	0.5	even	ok
485.2785	C22H45O9S	485.2790	0.9	17.6	0.5	even	ok
513.3105	C24H49O9S	513.3103	-0.5	30.3	0.5	even	ok
529.3049	C24H49O10S	529.3052	0.5	19.9	0.5	even	ok
557.3369	C26H53O10S	557.3365	-0.7	34.8	0.5	even	ok
573.3313	C26H53O11S	573.3314	0.2	25.3	0.5	even	ok
601.3635	C28H57O11S	601.3627	-1.2	39.8	0.5	even	ok
617.3581	C28H57O12S	617.3576	-0.8	33.1	0.5	even	ok
661.3847	C30H61O13S	661.3838	-1.3	38.2	0.5	even	ok
705.4110	C32H65O14S	705.4101	-1.4	71.6	0.5	even	ok
749.4375	C34H69O15S	749.4363	-1.7	69.3	0.5	even	ok

Fig. S22. Negative-ion ESI spectrum of dusy women Duschgel. ESI conditions: 6 µl min^{-1} of a solution at 0.1 µl ml^{-1} in methanol : water = 9 : 1. Yellow dots at the peak tops mark peaks with formula assignments in the list below the spectral plot.

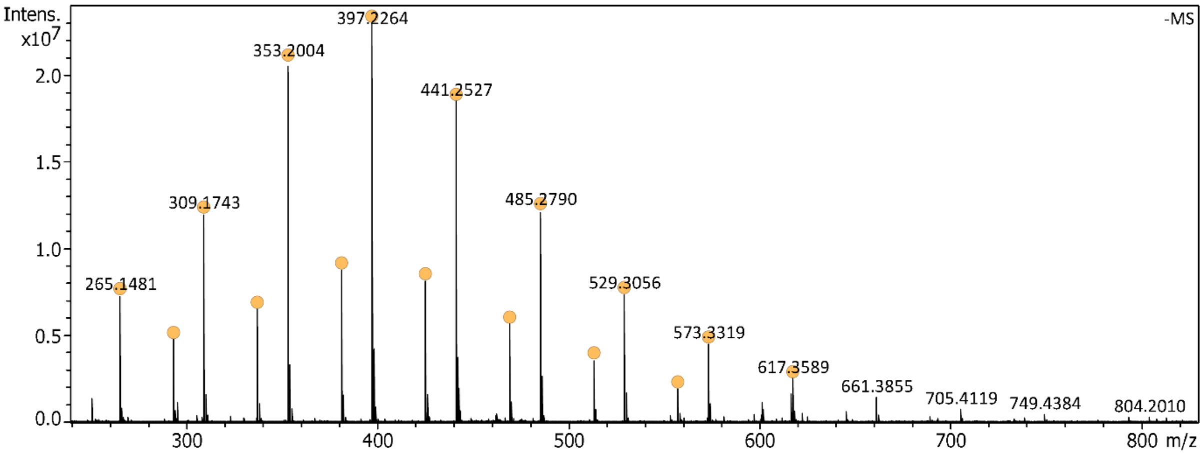

Meas. m/z	Ion Formula	m/z	err [ppm]	mSigma	rdb	e⁻ Conf	N-Rule
265.1481	C12H25O4S	265.1479	-0.9	8.4	0.5	even	ok
293.1795	C14H29O4S	293.1792	-1.0	7.8	0.5	even	ok
309.1743	C14H29O5S	309.1741	-0.7	9.5	0.5	even	ok
337.2058	C16H33O5S	337.2054	-1.0	11.8	0.5	even	ok
353.2004	C16H33O6S	353.2003	-0.1	9.7	0.5	even	ok
381.2320	C18H37O6S	381.2316	-0.9	12.1	0.5	even	ok
397.2264	C18H37O7S	397.2265	0.3	12.3	0.5	even	ok
425.2582	C20H41O7S	425.2578	-0.8	15.0	0.5	even	ok
441.2527	C20H41O8S	441.2528	0.1	18.8	0.5	even	ok
469.2846	C22H45O8S	469.2841	-1.2	22.5	0.5	even	ok
485.2790	C22H45O9S	485.2790	-0.1	21.1	0.5	even	ok
513.3110	C24H49O9S	513.3103	-1.4	28.4	0.5	even	ok
529.3056	C24H49O10S	529.3052	-0.7	24.2	0.5	even	ok
557.3376	C26H53O10S	557.3365	-2.0	21.1	0.5	even	ok
573.3319	C26H53O11S	573.3314	-0.9	36.6	0.5	even	ok
617.3589	C28H57O12S	617.3576	-2.1	32.3	0.5	even	ok

Fig. S23. Negative-ion APFD spectrum of Blasocut cooling lubricant. APFD conditions: 1–2 µl of a solution at 2 µl ml^{-1} in methanol : water = 9 : 1, acquisition 16 × 2.0 s, desolvation gas 1.2 l min^{-1} at 140 °C, shield 4.7 kV, cap 5.0 kV. Yellow dots at the peak tops mark peaks with formula assignments in the list below the spectral plot.

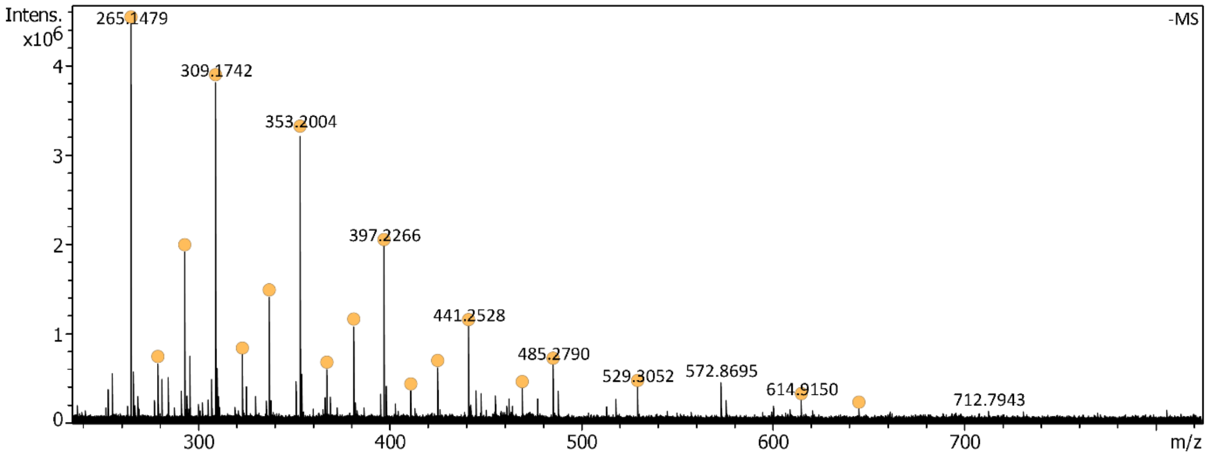

Meas. m/z	Ion Formula	m/z	err [ppm]	mSigma	rdb	e⁻ Conf	N-Rule
265.1479	C12H25O4S	265.1479	-0.1	6.8	0.5	even	ok
279.1273	C12H23O5S	279.1272	-0.3	n.a.	1.5	even	ok
293.1792	C14H29O4S	293.1792	-0.1	18.1	0.5	even	ok
309.1742	C14H29O5S	309.1741	-0.1	8.1	0.5	even	ok
323.1534	C14H27O6S	323.1534	-0.0	19.9	1.5	even	ok
337.2055	C16H33O5S	337.2054	-0.2	14.3	0.5	even	ok
353.2004	C16H33O6S	353.2003	-0.1	9.7	0.5	even	ok
367.1796	C16H31O7S	367.1796	-0.1	20.7	1.5	even	ok
381.2316	C18H37O6S	381.2316	0.1	25.8	0.5	even	ok
397.2266	C18H37O7S	397.2265	-0.0	23.9	0.5	even	ok
411.2058	C18H35O8S	411.2058	0.1	n.a.	3.0	even	ok
425.2579	C20H41O7S	425.2578	-0.2	26.9	0.5	even	ok
441.2528	C20H41O8S	441.2528	0.0	30.9	0.5	even	ok
469.2839	C22H45O8S	469.2841	0.3	32.9	0.5	even	ok
485.2790	C22H45O9S	485.2790	-0.1	70.3	2.0	even	ok
529.3052	C24H49O10S	529.3052	0.1	34.6	2.0	even	ok
614.9150	C27H3O16S	614.9147	-0.5	n.a.	28.0	even	ok
645.1521	C45H25O3S	645.1530	1.4	n.a.	35.0	even	ok

Fig. S24. Negative-ion ESI spectrum of Blasocut cooling lubricant. ESI conditions: 6 µl min^{-1} of a solution at 0.1 µl ml^{-1} in methanol : water = 9 : 1. Yellow dots at the peak tops mark peaks with formula assignments in the list below the spectral plot.

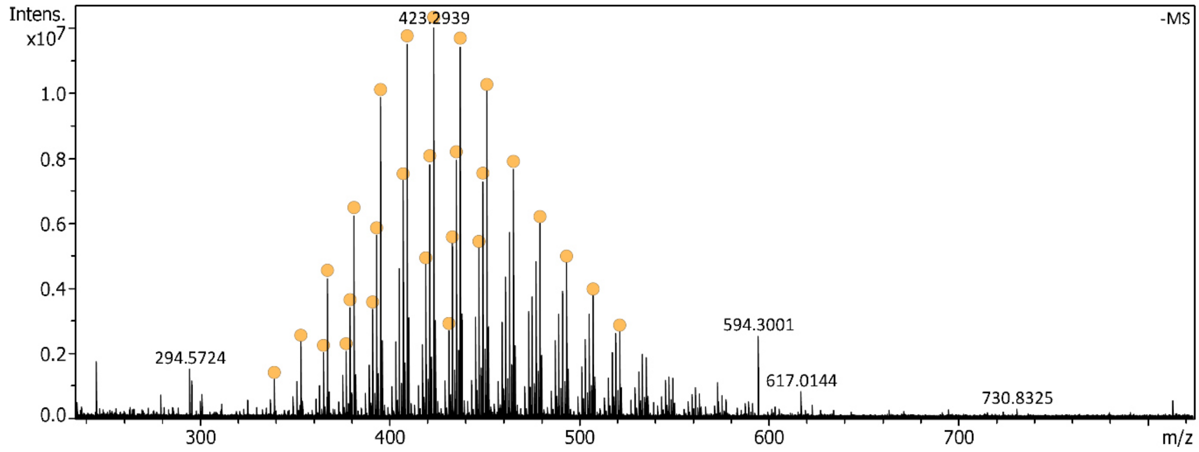

Meas. m/z	Ion Formula	m/z	err [ppm]	mSigma	rdb	e⁻ Conf	N-Rule
339.2000	C19H31O3S	339.1999	-0.1	19.3	4.5	even	ok
353.2156	C20H33O3S	353.2156	-0.1	20.7	4.5	even	ok
365.2157	C21H33O3S	365.2156	-0.2	27.4	5.5	even	ok
367.2313	C21H35O3S	367.2312	-0.2	18.7	4.5	even	ok
377.2156	C22H33O3S	377.2156	-0.1	14.2	6.5	even	ok
379.2313	C22H35O3S	379.2312	-0.1	12.3	5.5	even	ok
381.2469	C22H37O3S	381.2469	0.0	10.6	4.5	even	ok
391.2313	C23H35O3S	391.2312	-0.1	22.0	6.5	even	ok
393.2469	C23H37O3S	393.2469	-0.1	6.3	5.5	even	ok
395.2626	C23H39O3S	395.2625	-0.0	6.8	4.5	even	ok
407.2626	C24H39O3S	407.2625	-0.0	36.1	5.5	even	ok
409.2782	C24H41O3S	409.2782	0.0	5.6	4.5	even	ok
419.2626	C25H39O3S	419.2625	-0.2	15.3	6.5	even	ok
421.2782	C25H41O3S	421.2782	-0.1	18.6	5.5	even	ok
423.2939	C25H43O3S	423.2938	-0.0	9.2	4.5	even	ok
431.2626	C26H39O3S	431.2625	-0.1	19.0	7.5	even	ok
433.2782	C26H41O3S	433.2782	-0.1	16.1	6.5	even	ok
435.2939	C26H43O3S	435.2938	-0.1	17.6	5.5	even	ok
437.3095	C26H45O3S	437.3095	-0.1	6.6	4.5	even	ok
447.2939	C27H43O3S	447.2938	-0.2	379.3	6.5	even	ok
449.3096	C27H45O3S	449.3095	-0.1	353.3	5.5	even	ok
451.3252	C27H47O3S	451.3251	-0.0	7.4	4.5	even	ok
465.3408	C28H49O3S	465.3408	-0.0	7.2	4.5	even	ok
479.3565	C29H51O3S	479.3564	-0.1	6.7	4.5	even	ok
493.3722	C30H53O3S	493.3721	-0.2	6.4	4.5	even	ok
507.3879	C31H55O3S	507.3877	-0.2	20.7	4.5	even	ok
521.4035	C32H57O3S	521.4034	-0.2	8.5	4.5	even	ok

Fig. S25. Negative-ion APFD spectrum of Alufluid cooling lubricant. APFD conditions: 1–2 µl of a solution at 10 µl ml^{-1} in methanol : water = 9 : 1, acquisition 16 × 1.0 s, desolvation gas 1.0 l min^{-1} at 100 °C, shield 4.5 kV, cap 4.8 kV. Yellow dots at the peak tops mark peaks with formula assignments in the list below the spectral plot.

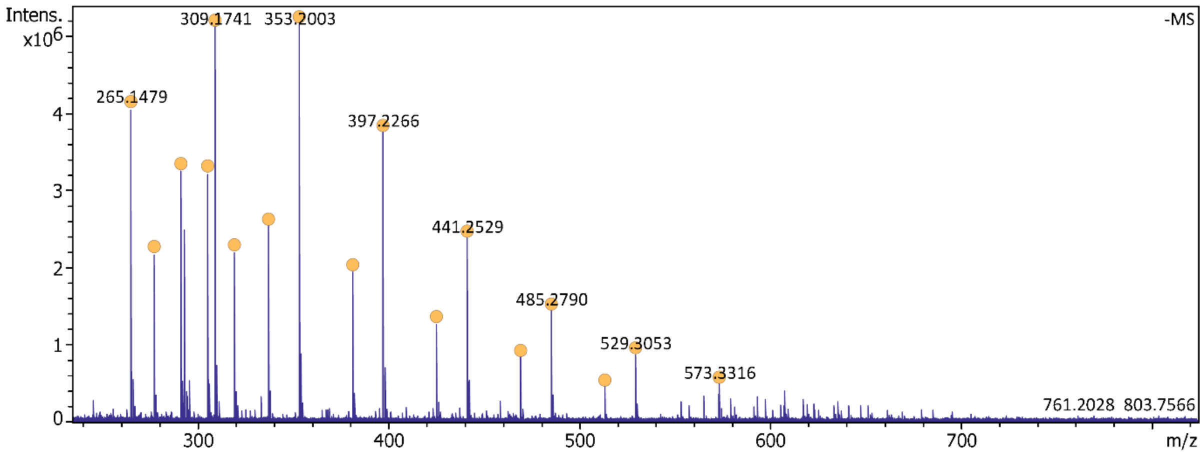

Meas. m/z	Ion Formula	m/z	err [ppm]	mSigma	rdb	e⁻ Conf	N-Rule
265.1479	C12H25O4S	265.1479	-0.0	9.3	0.5	even	ok
277.1843	C14H29O3S	277.1843	0.0	11.7	0.5	even	ok
291.1999	C15H31O3S	291.1999	0.1	4.2	0.5	even	ok
305.2156	C16H33O3S	305.2156	0.1	14.6	0.5	even	ok
309.1741	C14H29O5S	309.1741	0.1	7.8	0.5	even	ok
319.2312	C17H35O3S	319.2312	0.0	14.7	0.5	even	ok
337.2054	C16H33O5S	337.2054	-0.1	18.8	0.5	even	ok
353.2003	C16H33O6S	353.2003	-0.0	9.1	0.5	even	ok
381.2317	C18H37O6S	381.2316	-0.1	8.4	0.5	even	ok
397.2266	C18H37O7S	397.2265	-0.2	18.7	0.5	even	ok
425.2580	C20H41O7S	425.2578	-0.3	29.8	0.5	even	ok
441.2529	C20H41O8S	441.2528	-0.3	19.5	0.5	even	ok
469.2842	C22H45O8S	469.2841	-0.2	36.0	0.5	even	ok
485.2790	C22H45O9S	485.2790	0.0	23.8	0.5	even	ok
513.3104	C24H49O9S	513.3103	-0.2	54.1	0.5	even	ok
529.3053	C24H49O10S	529.3052	-0.2	26.8	0.5	even	ok
573.3316	C26H53O11S	573.3314	-0.4	36.6	0.5	even	ok

Fig. S26. Negative-ion ESI spectrum of Alufluid cooling lubricant. ESI conditions: 6 µl min^{-1} of a solution at 0.2 µl ml^{-1} in methanol : water = 9 : 1. Yellow dots at the peak tops mark peaks with formula assignments in the list below the spectral plot. In contrast to all previous samples, in Alufluid there are several carboxylates, too.

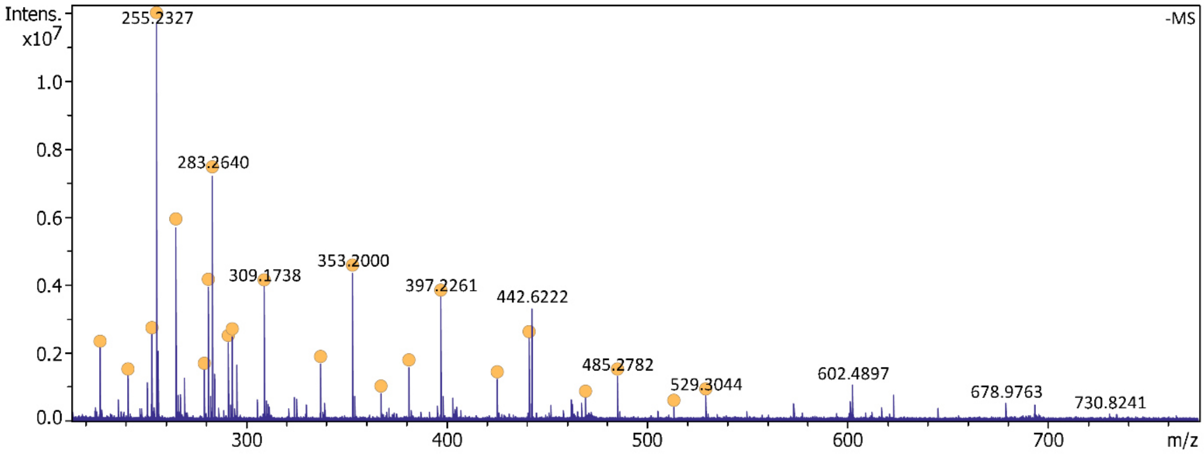

Meas. m/z	Ion Formula	m/z	err [ppm]	mSigma	rdb	e$^-$ Conf	N-Rule
227.2015	$C_{14}H_{27}O_2$	227.2017	0.8	6.7	1.5	even	ok
241.2171	$C_{15}H_{29}O_2$	241.2173	0.8	9.1	1.5	even	ok
253.2171	$C_{16}H_{29}O_2$	253.2173	0.8	11.4	2.5	even	ok
255.2327	$C_{16}H_{31}O_2$	255.2330	0.9	1.9	1.5	even	ok
265.1477	$C_{12}H_{25}O_4S$	265.1479	0.9	6.7	0.5	even	ok
279.1633	$C_{13}H_{27}O_4S$	279.1636	0.9	20.2	0.5	even	ok
281.2484	$C_{18}H_{33}O_2$	281.2486	0.9	7.4	2.5	even	ok
283.2640	$C_{18}H_{35}O_2$	283.2643	0.9	2.7	1.5	even	ok
291.1963	$C_{18}H_{27}O_3$	291.1966	0.9	13.7	5.5	even	ok
293.1789	$C_{14}H_{29}O_4S$	293.1792	1.0	19.5	0.5	even	ok
309.1738	$C_{14}H_{29}O_5S$	309.1741	0.9	8.6	0.5	even	ok
337.2051	$C_{16}H_{33}O_5S$	337.2054	0.9	115.8	0.5	even	ok
353.2000	$C_{16}H_{33}O_6S$	353.2003	1.0	12.6	0.5	even	ok
367.3578	$C_{24}H_{47}O_2$	367.3582	0.9	23.0	1.5	even	ok
381.2312	$C_{18}H_{37}O_6S$	381.2316	1.1	25.0	0.5	even	ok
397.2261	$C_{18}H_{37}O_7S$	397.2265	1.2	15.0	0.5	even	ok
425.2573	$C_{20}H_{41}O_7S$	425.2578	1.3	26.7	0.5	even	ok
441.2522	$C_{20}H_{41}O_8S$	441.2528	1.3	21.0	0.5	even	ok
469.2834	$C_{22}H_{45}O_8S$	469.2841	1.4	105.1	0.5	even	ok
485.2782	$C_{22}H_{45}O_9S$	485.2790	1.5	33.2	0.5	even	ok
513.3094	$C_{24}H_{49}O_9S$	513.3103	1.6	n.a.	0.5	even	ok
529.3044	$C_{24}H_{49}O_{10}S$	529.3052	1.5	33.7	0.5	even	ok

Fig. S27. Negative-ion APFD spectrum of dusy women Duschgel. APFD conditions: 1 µl of a solution in methanol : water = 9 : 1, acquisition 16 × 1.5 s, desolvation gas 1.2 l min^{-1} at 140 °C, shield 3.8 kV, cap 4.3 kV. Spectra acquired **a)** and **b)** at 1.0 µl ml^{-1}, **c)** and **d)** at 0.1 µl ml^{-1}, and **e)** and **f)** at 0.01 µl ml^{-1}. While **a)** to **d)** deliver good spectra, **e)** and **f)** only show a background with some noise signals (cf. Fig. S6).

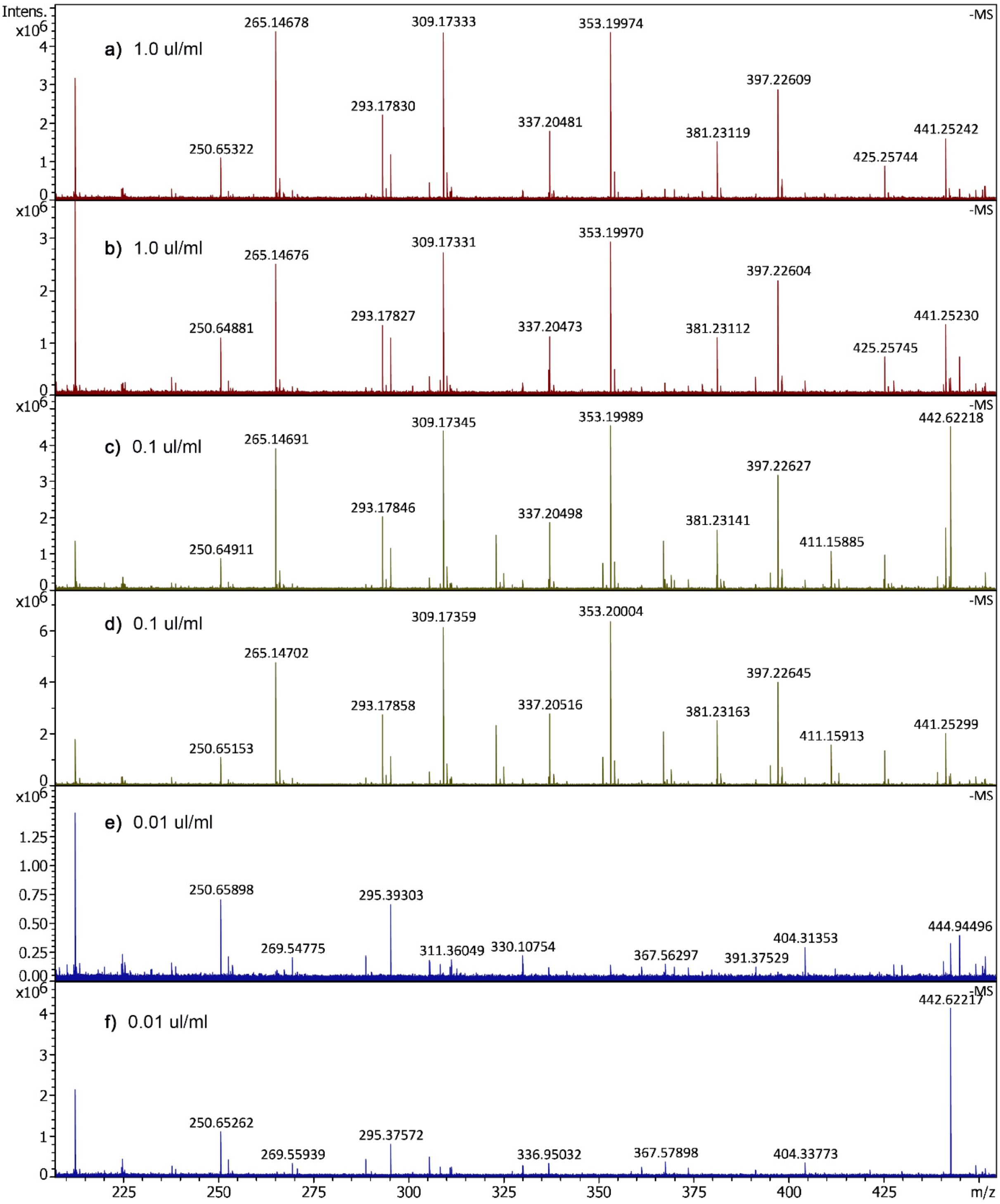

Supplement: Supplementary file 1 — Supplementary file1 (PDF 10396 KB) [file 216_2023_4917_MOESM1_ESM.pdf]
